# Cellular Proliferation and Reprogramming in Pre‐Metastatic Niches: Mechanisms and Therapeutic Vulnerabilities

**DOI:** 10.1111/cpr.70296

**Published:** 2026-11-10

**Authors:** Shengqian Wang, Chenghu Song, Pengfei Li, Junjie Feng, Xiaoxuan Shi, Qiwen Lu, Bohan Duan, Xinyan Zhang, Siqi Li, Yang Luo, Xiaohui Fan, Ziyun Li, Yang Zhang, Renjun Gu, Wenjun Mao

**Affiliations:** ^1^ First Clinical Medical College, Nanjing University of Chinese Medicine Nanjing China; ^2^ Department of Thoracic Surgery The Affiliated Wuxi People's Hospital of Nanjing Medical University, Wuxi People's Hospital, Wuxi Medical Center, Nanjing Medical University Wuxi China; ^3^ Department of Clinical Laboratory Jiangsu Province Hospital of Chinese Medicine, Affiliated Hospital of Nanjing University of Chinese Medicine Nanjing China; ^4^ College of Integrated Traditional Chinese and Western Medicine, Nanjing University of Chinese Medicine Nanjing China; ^5^ School of Chinese Medicine, Nanjing University of Chinese Medicine Nanjing China; ^6^ Department of Laboratory Medicine Chongqing Center for Clinical Laboratory, Chongqing Academy of Medical Sciences, Chongqing General Hospital, School of Medicine, Chongqing University Chongqing China; ^7^ State Key Laboratory of Chinese Medicine Modernization, College of Pharmaceutical Sciences, Zhejiang University Hangzhou China; ^8^ School of Pharmaceutical Sciences, Zhejiang Chinese Medical University Hangzhou China; ^9^ School of Acupuncture and Tuina, School of Regimen and Rehabilitation Nanjing University of Chinese Medicine Nanjing China; ^10^ Department of Rehabilitation Medicine, School of Acupuncture‐Moxibustion and Tuina and School of Health Preservation and Rehabilitation Nanjing University of Chinese Medicine Nanjing China; ^11^ Department of Gastroenterology and Hepatology Jinling Hospital, Medical School of Nanjing University Nanjing China

**Keywords:** cellular reprogramming, disseminated tumour cell dormancy, metabolic reprogramming, microenvironment normalization, pre‐metastatic niche, spatial omics

## Abstract

Cancer metastasis, the leading cause of cancer‐related mortality, fundamentally depends on the proactive remodelling of distant organ microenvironments by primary tumours through systemic signals, creating a pre‐metastatic niche before tumour cell arrival. This review systematically deconstructs the dynamic spatiotemporal evolution of the pre‐metastatic niche, highlighting the multi‐layered reprogramming of stromal, endothelial and immune cells that collectively cultivate a pro‐tumourigenic and immunosuppressive microenvironment. We elucidate the biomechanical and biochemical sensing mechanisms, derived primarily from experimental models, that dictate the fate of disseminated tumour cells, governing the critical balance between dormancy maintenance and proliferative reactivation. Furthermore, we synthesize mechanistic insights alongside human tissue and liquid‐biopsy correlative evidence on how metabolic resetting and epigenetic memory may contribute to sustaining pre‐metastatic niche stability, and discuss how cutting‐edge single‐cell and spatial omics technologies are deciphering the intricate multicellular topological architecture of the niche. Ultimately, by mapping these complex systemic interactions across preclinical, biomarker and translational levels, we identify potential therapeutic vulnerabilities and advocate for a paradigm shift toward microenvironment normalization. While clinical validation remains an evolving frontier, targeting pre‐metastatic niche priming represents a promising window that warrants rigorous clinical evaluation to intercept nascent metastasis before clinically detectable colonization occurs.

## Graphical abstract

This review systematically elucidates how primary tumours remotely drive multicellular reprogramming to construct the pre‐metastatic niche and dictate disseminated tumour cell fate. By mapping this dynamic ecosystem, we identify actionable therapeutic vulnerabilities, advocating a paradigm shift from traditional cytotoxicity toward microenvironment normalization for early metastasis interception.

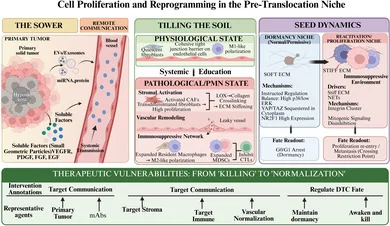

**Abbreviations**
AI: artificial intelligence
ARG1: arginase‐1
ATRA: all‐trans retinoic acid
BMP: bone morphogenetic protein
CAF: cancer‐associated fibroblast
CCL: chemokine (C‐C motif) ligand
CCND1: cyclin D1
CCR2: C‐C chemokine receptor type 2
CDK: cyclin‐dependent kinase
cDNA: complementary DNA
CRISPR: clustered regularly interspaced short palindromic repeats
CSF‐1R: colony‐stimulating factor 1 receptor
CTCs: circulating tumour cells
CTLA‐4: cytotoxic T‐lymphocyte‐associated protein 4
CXCL: C‐X‐C motif chemokine ligand
CXCR4: C‐X‐C chemokine receptor type 4
DNase: deoxyribonuclease
DTCs: disseminated tumour cells
ECM: extracellular matrix
EMT: epithelial–mesenchymal transition
ERK: extracellular signal‐regulated kinase
EVs: extracellular vesicles
EZH2: enhancer of zeste homologue 2
FAK: focal adhesion kinase
G‐CSF: granulocyte colony‐stimulating factor
GSN: gelsolin
H3K27me3: trimethylation of histone H3 at lysine 27
H3K4me3: trimethylation of histone H3 at lysine 4
HIF‐1α: hypoxia‐inducible factor 1‐alpha
HSCs: hepatic stellate cells
HSPC111: haematopoietic stem/progenitor cell protein 111
ICIs: immune checkpoint inhibitors
IFP: interstitial fluid pressure
IL: interleukin
iNOS: inducible nitric oxide synthase
LOX: lysyl oxidase
MAPK: mitogen‐activated protein kinase
MCP‐1: monocyte chemoattractant protein‐1
MDSCs: myeloid‐derived suppressor cells
MERFISH: multiplexed error‐robust fluorescence in situ hybridization
MHC: major histocompatibility complex
miRNAs: microRNAs
MMPs: matrix metalloproteinases
MRD: minimal residual disease
MSCs: mesenchymal stem cells
MVBs: multivesicular bodies
ncRNAs: non‐coding RNAs
NF‐κB: nuclear factor kappa‐light‐chain‐enhancer of activated B cells
NK cells: natural killer cells
NR2F1: nuclear receptor subfamily 2, group F, member 1
p38 MAPK: p38 mitogen‐activated protein kinase
PAD4: peptidyl arginine deiminase 4
PD‐1: programmed cell death protein 1
PD‐L1: programmed death‐ligand 1
PI3K: phosphoinositide 3‐kinase
PlGF: placental growth factor
PMN: pre‐metastatic niches
Rb: retinoblastoma protein
RNA: ribonucleic acid
ROS: reactive oxygen species
S100A8/A9: S100 calcium‐binding protein A8/A9
scRNA‐seq: single‐cell RNA sequencing
SDF‐1: stromal cell‐derived factor 1
SMAD: mothers against decapentaplegic homologue
SMYD3: SET and MYND domain‐containing protein 3
STAT3: signal transducer and activator of transcription 3
TAMs: tumour‐associated macrophages
TAZ: transcriptional coactivator with PDZ‐binding motif
TEPs: tumour‐educated platelets
TGF‐β: transforming growth factor‐beta
TME: tumour microenvironment
TNF‐α: tumour necrosis factor‐alpha
Tregs: regulatory T cells
VCAM‐1: vascular cell adhesion molecule 1
VEGF: vascular endothelial growth factor
VEGFR1: vascular endothelial growth factor receptor 1
YAP: Yes‐associated protein

## 1 Introduction

Cancer metastasis remains the leading cause of death in patients with solid tumours. Its initiation and progression depend not only on the properties of tumour cells themselves but also on complex systemic interactions between disseminated tumour cells and the host microenvironment [1, 2, 3]. More than a century ago, Paget proposed the classical Seed and Soil hypothesis, which has since guided much of the thinking on organotropic metastasis [4, 5, 6]. This hypothesis emphasizes that disseminated tumour cells passively adapt to the specific microenvironments of distant organs. However, this traditional view is largely static and descriptive. It does not adequately explain how the soil is actively remodelled and acquires protumourigenic features before tumour cells actually arrive [7, 8].

With advances in tumour systems biology, the concept of the premetastatic niche has challenged this old paradigm and shifted the research focus from passive acceptance of the soil to active remote construction by the primary tumour [6, 8]. The premetastatic niche is not a preexisting anatomical structure in distant organs. Instead, it is a pathological microenvironment that the primary tumour prepares across an organ scale [9]. Through continuous release of systemic signals, such as extracellular vesicles and soluble factors, the primary tumour remotely drives phenotypic reprogramming of resident cells in target organs, inducing local inflammation, microvascular leakage and immune evasion [10, 11, 12]. As a result, this process creates a favourable microenvironment for the subsequent colonization and expansion of malignant clones.

Based on the dynamic spatiotemporal evolution of the premetastatic niche, this review takes the concept of tilling before sowing as a starting point to systematically dissect the earliest steps of the metastatic cascade. Current evidence suggests that successful organ‐specific metastasis depends not only on the invasive genetic evolution of malignant cells but also, and more importantly, on the remote education that the primary tumour imposes on the distant target organ microenvironment [8, 9, 13, 14, 15]. In this systemic remodelling network, the responses of resident cells in target organs are not merely accompanying pathological changes. Rather, these cells undergo a multilayered reprogramming process involving cell cycle derepression, phenotypic transitions, metabolic resetting and epigenetic memory [16, 17, 18, 19]. Such profound changes in cellular states represent a critical rate‐limiting step that closes the loop of the metastatic cascade.

Therefore, moving the focus of metastasis research to the premetastatic niche, a controllable window before clinically detectable metastasis forms, not only helps to reunderstand the systemic nature of cancer from a systems biology perspective but also provides important theoretical support and forward‐looking therapeutic strategies for intercepting early colonization. This review will concentrate on the topological structure of multicellular interactions within the premetastatic niche, the cell cycle sensing mechanisms that regulate dormant disseminated tumour cells and their reactivation, the basic driving logic of metabolism and epigenetics and recent advances in single‐cell and spatial omics technologies for resolving niche heterogeneity. Finally, we explore vulnerabilities that disrupt the systemic stability of the premetastatic niche from the viewpoint of microenvironment normalization, aiming to provide new insights that push clinical antimetastatic interventions from reactive treatment of established disease toward proactive prevention of nascent metastasis.

## 2 The Architects of Proliferation: Systemic Signalling and Communication

### 2.1 Tumour‐Derived Extracellular Vesicles: Signalling, Tropism and Methodological Considerations

Tumour‐derived EVs, encompassing heterogeneous populations of membrane‐bound vesicles such as small EVs and microvesicles, serve as key systemic drivers of long‐range pro‐metastatic communication in specific tumour contexts [20, 21]. Upon internalization by resident stromal, endothelial or immune cells within distant target organs, tumour EVs deliver functional biomolecular cargo, including specific microRNAs, non‐coding RNAs, signalling proteins and lipids that induce local proliferation and phenotypic reprogramming in a tissue‐dependent manner [11, 22].

Crucially, these signalling mechanisms reflect specialized pathways identified in specific experimental models rather than universal rules across all malignancies. For instance, in syngeneic murine models of triple‐negative breast cancer (4T1), tumour‐derived EVs were shown to transport latency‐associated peptide‐TGF‐β1 or Wnt7a to pulmonary fibroblasts and endothelial cells, inducing fibroblast transdifferentiation into cancer‐associated fibroblasts and driving extracellular matrix remodelling to promote lung‐specific metastatic seeding [23, 24, 25, 26]. Similarly, receptor‐mediated organotropic targeting exhibits strict tumour‐ and tissue‐specificity. Seminal primary studies in breast cancer and melanoma models demonstrated that surface integrin heterodimers on tumour EVs act as organotropic zip codes, where EV‐bound integrins α6β4 and α6β1 selectively bind pulmonary laminin to foster lung tropism, whereas integrin αvβ5 binds hepatic fibronectin to promote liver tropism [11, 24, 27, 28]. In pancreatic adenocarcinoma models, CD44v6 expression on tumour EVs was specifically implicated in facilitating EV uptake by soluble ligand‐bearing stromal cells in lymph node and liver microenvironments [29, 30, 31, 32]. Because these molecular zip‐codes and signalling cascades were delineated in specific cancer cell lines and animal models, caution must be exercised when extrapolating these mechanisms as universal principles across all human solid tumours or metastatic organs.

### 2.2 Soluble Factors: Cytokines and Chemokines as Messengers

Beyond extracellular vesicles, various soluble factors secreted by the primary tumour can systemically disrupt host homeostasis. Vascular endothelial growth factor, placental growth factor and transforming growth factor‐beta are upregulated in many types of malignancies [33, 34, 35, 36]. They drive pre‐metastatic niche formation by promoting endothelial cell proliferation, increasing vascular permeability and compromising vascular barrier integrity [17, 37]. Pro‐inflammatory cytokines such as tumour necrosis factor‐alpha and interleukin‐1‐beta induce the expression of chemokines like CXCL1, thereby amplifying systemic inflammation and recruiting myeloid cells [38, 39]. Moreover, cytokines like granulocyte colony‐stimulating factor mobilize and expand granulocytic myeloid‐derived suppressor cells [40, 41]. These cells infiltrate target organs under chemotactic guidance and contribute to the establishment of an immunosuppressive microenvironment [38, 42].

### 2.3 Routes of Transmission: From Local Secretion to Distant Deposition

These systemic signals cross tissue and bloodstream barriers to accumulate in specific target organs [43]. Guided by tumour‐secreted chemotactic signals such as stromal cell‐derived factor 1 and S100A8/A9, vascular endothelial growth factor receptor 1‐positive haematopoietic progenitor cells and myeloid‐derived suppressor cells are recruited to pre‐metastatic sites [6, 15, 44]. Once there, these cells undergo local expansion regulated by transcription factors such as NF‐κB and STAT3 and secrete matrix metalloproteinases that degrade the extracellular matrix and release local growth factors [45, 46]. This process works together with tumour‐associated hypoxic responses, including the upregulation of hypoxia‐inducible factor 1‐alpha, to drive the formation of angiogenic and immunosuppressive niches in a spatiotemporally coordinated manner [47, 48]. In summary, these systemic signals remodel the target organ microenvironment through a dynamic spatiotemporal cascade. The overall framework of this process involves three distinct stages, a systemic signalling network and local transitions in cellular states (Figure 1). The specific signalling molecules secreted by the primary tumour to drive distant niche construction, along with their induced proliferative or reprogramming effects, are summarized in Table 1.

**FIGURE 1:**
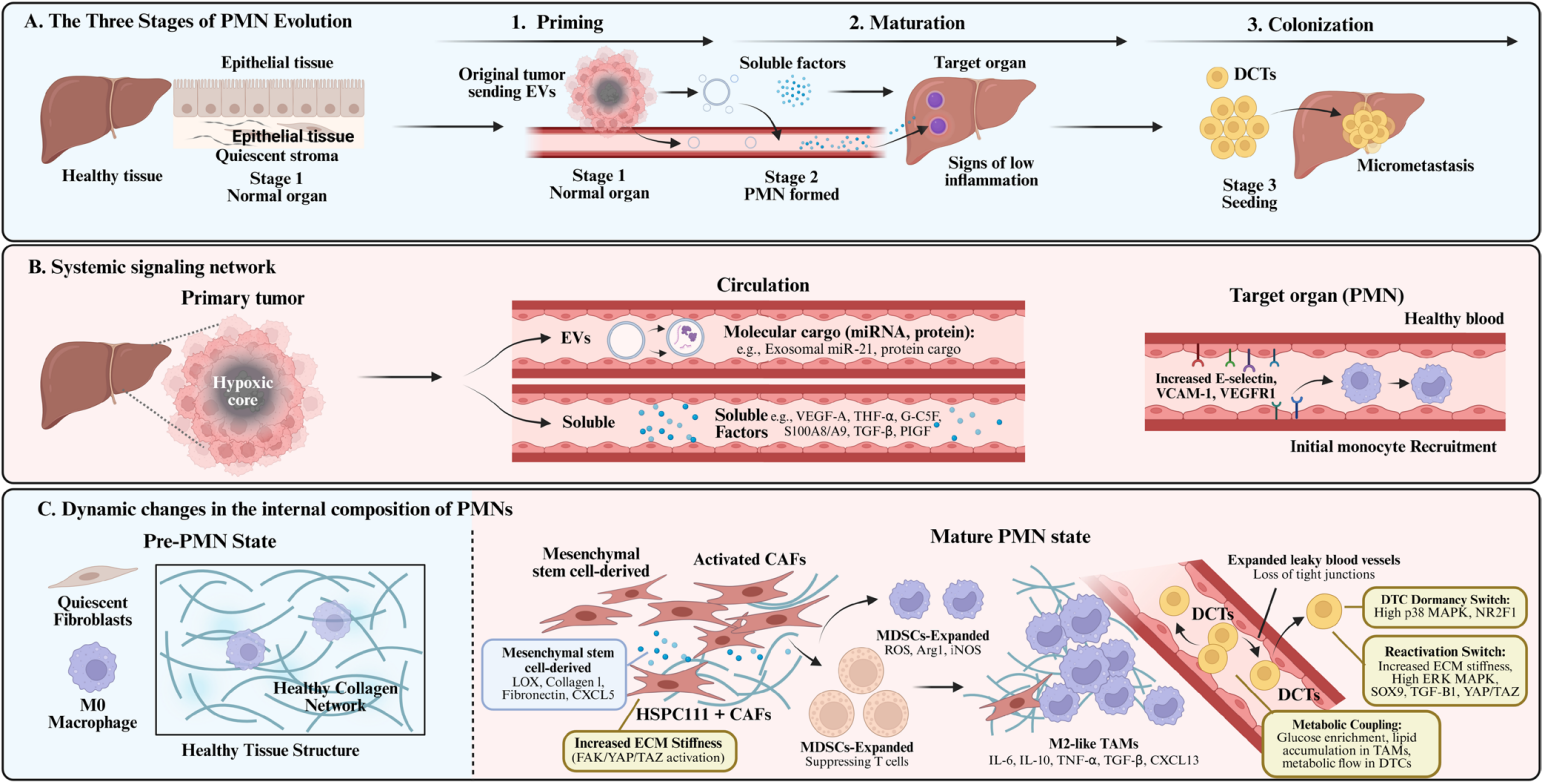
Spatiotemporal dynamics of pre‐metastatic niche formation and multi‐cellular remodelling. The schematic illustrates the three stages of pre‐metastatic niche evolution, the systemic signalling network and the local transition from a quiescent to a mature pre‐metastatic niche state characterized by stromal activation, immune suppression and extracellular matrix remodelling. Created in BioRender. Xiao, H. (2026) https://BioRender.com/z0o1w56.

**TABLE 1:** Key systemic signalling molecules and their induced proliferative and reprogramming effects in the pre‐metastatic niche.

Systemic signal	Source	Targeted distal cell types	Proliferation/reprogramming effect	References
Exosomal miR‐21	Lung cancer cells	Lung fibroblasts	Induce the proliferation of fibroblasts and their transdifferentiation into tumour‐associated fibroblasts, promoting the formation of fibrotic niches.	[10]
VEGF‐A	Primary tumour cells	Endothelial cells	Promote endothelial cell proliferation, increase vascular permeability, disrupt the blood–brain/vascular barrier and facilitate metastasis and spread.	[17, 37]
TNF‐α	Macrophages and T cells in the microenvironment of the primary tumour	Endothelial cells	Up‐regulate the expression of CXCL1 in HSCs, induce their proliferation and activation into myofibroblasts and drive the formation of pre‐metastasis niches in the liver.	[42]
G‐CSF	Primary tumour cells	Myeloid progenitor cells in the bone marrow	Mobilize and expand granulocytic myeloid‐derived suppressor cells, leading to their aggregation in the lungs before metastasis and the establishment of immunosuppressive niches.	[49]
S100A8/A9	Myeloid cells recruited to the pre‐metastasis site	Pre‐transfer niche stromal cells	Induce the expression of SAA3 and serum amyloid A, drive NF‐κ B‐dependent proliferation and inflammatory reprogramming of pulmonary stromal cells and enhance metastasis and colonization.	[50]
TGF‐β	Primary tumour cells (breast cancer, lung cancer)	Fibroblasts in target organs	Induce the expression of SAA3 and serum amyloid A, drive NF‐κ B‐dependent proliferation and inflammatory reprogramming of pulmonary stromal cells and enhance metastasis and colonization.	[50]

## 3 The Educated Soil: Cellular Reprogramming in Target Organs

Under the influence of systemic signalling, the local microenvironment of the target organ undergoes multidimensional functional remodelling, primarily involving the activation of stromal fibroblasts and extracellular matrix stiffening [51], the establishment of an immunosuppressive network [48], the disruption of the vascular barrier [11] and the phenotypic transformation of host parenchymal cells [20] (Figure 2).

**FIGURE 2:**
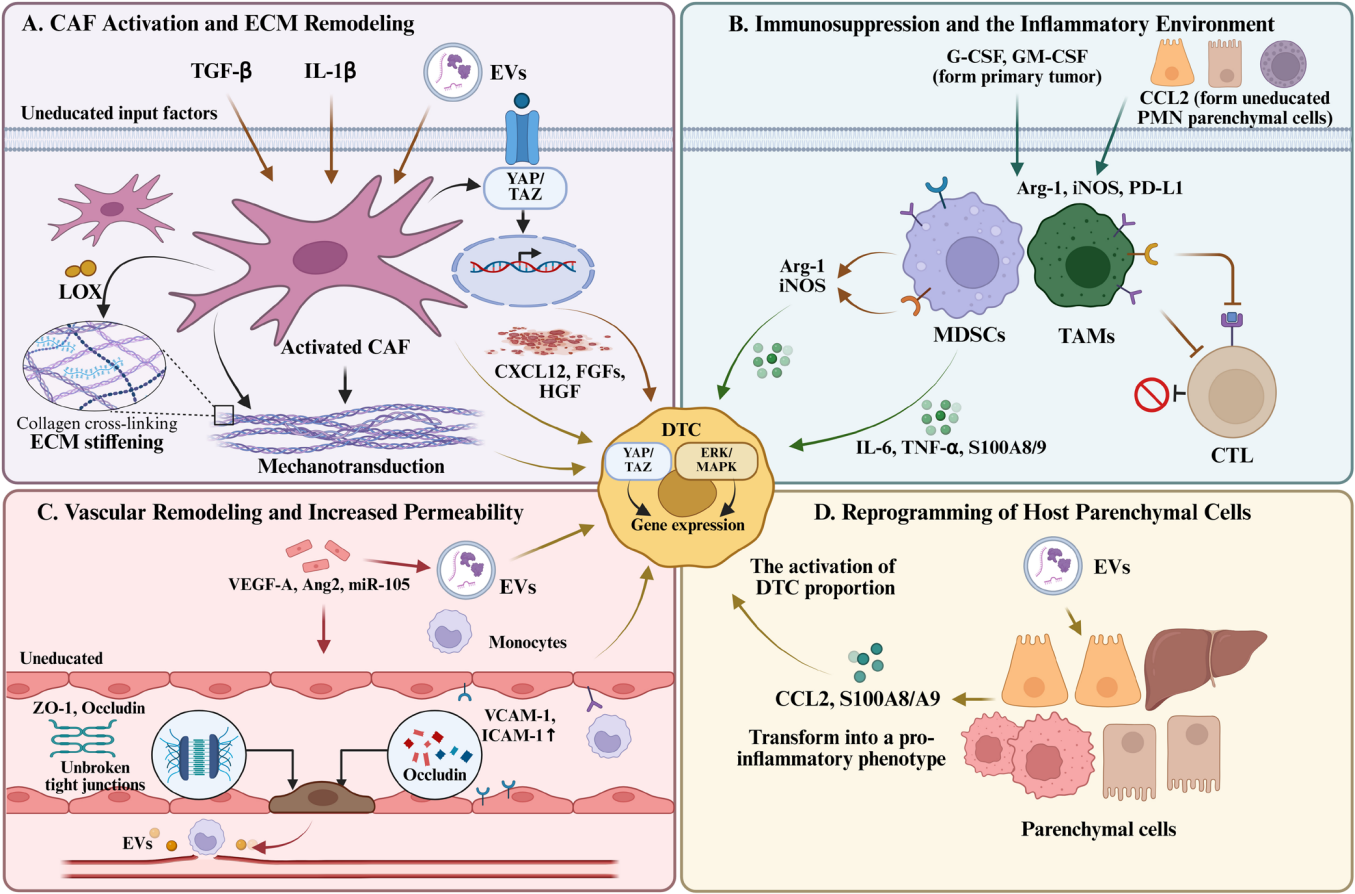
Key remodelling events within the target organ during pre‐metastatic niche formation. Tumour‐derived extracellular vesicles and soluble factors drive four interconnected processes: (A) activation of CAFs and extracellular matrix stiffening via LOX‐mediated collagen cross‐linking; (B) establishment of an immunosuppressive network through MDSC/TAM expansion and CTL suppression; (C) vascular hyperpermeability caused by disruption of tight junctions and upregulation of adhesion molecules; and (D) reprogramming of parenchymal cells toward a pro‐inflammatory phenotype that facilitates DTC colonization. Created in BioRender. Xiao, H. (2026) https://BioRender.com/z0o1w56.

### 3.1 Stromal Proliferative Activation

#### 3.1.1 Mesenchymal Stem Cells and Fibroblasts

Fibroblasts and mesenchymal stem cells are the primary effectors of stromal remodelling within the pre‐metastatic niche [52, 53]. Upon induction by tumour‐derived signals, such as extracellular vesicle‐delivered transforming growth factor beta, resident fibroblasts are reprogrammed into cancer‐associated fibroblasts and undergo proliferative expansion [54]. These cancer‐associated fibroblasts augment matrix stiffness by secreting lysyl oxidase, collagen and fibronectin, thereby forming a physical scaffold that favours tumour cell adhesion and survival [52, 55]. In addition, in colorectal cancer liver metastasis models, primary tumour‐derived vesicular HSPC111 remodels lipid metabolism in cancer‐associated fibroblasts and induces robust CXCL5 expression, further altering the local microenvironment [56].

Beyond extracellular matrix remodelling, cancer‐associated fibroblasts also exert immunomodulatory functions that shape the pre‐metastatic niche [52, 57]. Pan‐cancer single‐cell and spatial transcriptomic analyses have identified a subset of inflammatory cancer‐associated fibroblasts marked by gelsolin, whose expression is associated with resistance to immune checkpoint inhibitor therapy [58]. Mechanistically, gelsolin downregulation in cancer‐associated fibroblasts inactivates NF‐κB signalling, reduces pro‐inflammatory cytokine secretion and drives CD8+ T cells toward exhaustion and dysfunction [59]. Spatial transcriptomics further revealed that inflammatory cancer‐associated fibroblasts are located closer to CD8+ exhausted T cells in inhibitor non‐responders, suggesting that physical interactions within the pre‐metastatic niche facilitate immune evasion [60]. Importantly, restoring gelsolin expression in cancer‐associated fibroblasts enhanced anti‐PD‐1 efficacy in preclinical models [61, 62].

#### 3.1.2 Endothelial Cells

Endothelial cell proliferation and elevated vascular permeability are critical structural events during pre‐metastatic niche formation [63]. The synergistic action of vascular endothelial growth factor and matrix metalloproteinases not only promotes local angiogenesis but also disrupts the established endothelial barrier, resulting in vascular leakage [64]. For example, EVs secreted by renal cell carcinoma can induce pulmonary vascular hyperpermeability and basement membrane degradation, thereby creating a conduit for subsequent tumour cell extravasation.

### 3.2 The Immune Suppression Network

#### 3.2.1 Macrophage Polarization and Expansion

The polarization of resident macrophages within target organs is a critical step in establishing an immune‐privileged microenvironment [65]. Modulated by cancer‐associated fibroblast‐derived lactic acid and tumour‐derived EVs, these macrophages undergo a phenotypic transition from an anti‐tumour state to a pro‐tumourigenic M2‐like state [11, 66].

Beyond biochemical cues, physical signals from the remodelling extracellular matrix also directly influence macrophage fate [67, 68]. Increased matrix stiffness, a hallmark of the maturing pre‐metastatic niche driven by cancer‐associated fibroblast‐mediated collagen crosslinking, promotes pro‐inflammatory M1 polarization via the Piezo1‐YAP mechanotransduction axis [69, 70], whereas soft or intermediate stiffness favours the anti‐inflammatory M2 phenotype. Although the pre‐metastatic niche is typically M2‐dominant, these findings reveal that mechanical signals alone can instruct macrophage phenotype, suggesting that within the mechanically heterogeneous pre‐metastatic niche, macrophages may adopt distinct states depending on local stiffness [71]. This mechanical reprogramming complements chemical signals to shape the overall immune landscape. Subsequently, the polarized macrophages secrete factors such as CXCL13, establishing a positive feedback loop that continuously amplifies local immunosuppression [72].

#### 3.2.2 Myeloid‐Derived Suppressor Cells

The distant recruitment and expansion of bone marrow‐derived cells constitute a vital component of the immunosuppressive barrier within the pre‐metastatic niche [53]. In synergy with M2‐like macrophages, expanded myeloid‐derived suppressor cells inhibit the effector functions of cytotoxic T lymphocytes and natural killer cells through mechanisms such as amino acid depletion and the production of reactive oxygen species [73]. This coordinated suppression induces local immune anergy in the target organ, thereby providing a relatively secure haven for disseminated tumour cells [73].

### 3.3 The Spatial Architecture of the Niche: Topological Organization

The formation of the pre‐metastatic niche involves not only phenotypic alterations within single‐cell subsets but also the orderly assembly and topological reconstruction of multicellular communities in three‐dimensional space. Emerging high‐resolution technologies, such as spatial transcriptomics, have revealed that the pre‐metastatic niche is not a random mixture of cells; rather, it represents a highly complex ecosystem organized by spatially constrained ligand‐receptor interaction networks [74, 75].

During the initial establishment of this spatial architecture, fibroblasts and their derived cancer‐associated fibroblasts serve as the central structural scaffold [19, 76]. By secreting collagen, fibronectin and lysyl oxidase, cancer‐associated fibroblasts significantly augment matrix stiffness, laying the physical foundation for pre‐metastatic niche development [77, 78]. This matrix remodelling alters local biomechanical properties and provides structural anchors for the spatial positioning of multicellular communities [79]. Concurrently, elevated endothelial permeability and basement membrane degradation dismantle existing physical barriers, creating spatial conduits for the enrichment of circulating factors and subsequent tumour cell extravasation [10, 17, 80].

Building upon these physical scaffolds and conduits, functionally specialized spatial micro‐niches are progressively formed within the pre‐metastatic niche [10, 81]. For instance, stClinic, a dynamic graph model for integrating spatial multimodal data, has successfully identified localized niches formed by distinct cellular spatial aggregations [82]. These include malignant niches characterized by the enrichment of tumour‐associated macrophages, as well as B cell‐rich niches associated with favourable prognoses [82, 83]. These findings indicate that the aggregation of immunosuppressive networks at specific spatial coordinates may serve as a major driver of the pro‐metastatic function of the pre‐metastatic niche [9, 17, 84]. Integrating spatial topological organization into the conceptual framework of the educated soil provides a more comprehensive understanding of how target organ microenvironments are systemically remodelled [9]. Modulated by systemic signals, resident cell types within target organs undergo profound phenotypic transitions and functional reprogramming [14, 17, 77, 85, 86]. Their shift from a physiological baseline to a pro‐tumourigenic pre‐metastatic niche state is summarized in Table 2.

**TABLE 2:** Comparison of physiological and reprogrammed states of resident cells within the pre‐metastatic niche.

Cell type	Physiological state	Pre‐Metastatic Niche state (reprogrammed)	Key secretory factors and functional changes	References
Stromal cells	Quiescent Fibroblasts: Low turnover, maintain extracellular matrix homeostasis.	Activated CAFs: High proliferative rate, metabolic reprogramming (glycolysis).	LOX, TGF‐β, CXCL5, Collagen I. Remodel extracellular matrix stiffness to facilitate tumour anchoring.	[78]
Endothelial cells	Cohesive barrier: low permeability, anti‐thrombotic surface.	Activated and leaky: proliferative angiogenesis, loss of tight junctions.	VEGFA, MMP‐9, VCAM‐1. Promote vascular leakiness for tumour extravasation.	[87]
Macrophages	M1‐like (Classical): pro‐inflammatory, tumouricidal.	M2‐like (TAMs): immunosuppressive polarization, proliferative expansion.	IL‐10, TGF‐β, Arginase‐1. Create an immune‐privileged niche to evade surveillance.	[88]
Myeloid Progenitors	Differentiation in BM: Controlled haematopoiesis.	MDSCs (Expanded): Mobilized from bone marrow, systemic expansion.	ROS, ARG1, iNOS. Suppress T cell function and induce T cell apoptosis.	[89]

## 4 Seed Dynamics: Cell Cycle Control in Disseminated Tumour Cells

The fate of disseminated tumour cells in target organs is precisely regulated by microenvironmental signals [90, 91] and their transition between dormancy maintenance and proliferation reactivation depends on synergistic changes in mechanosensing [92], kinase signalling balance and core cell cycle mechanisms [93, 94] (Figure 3).

**FIGURE 3:**
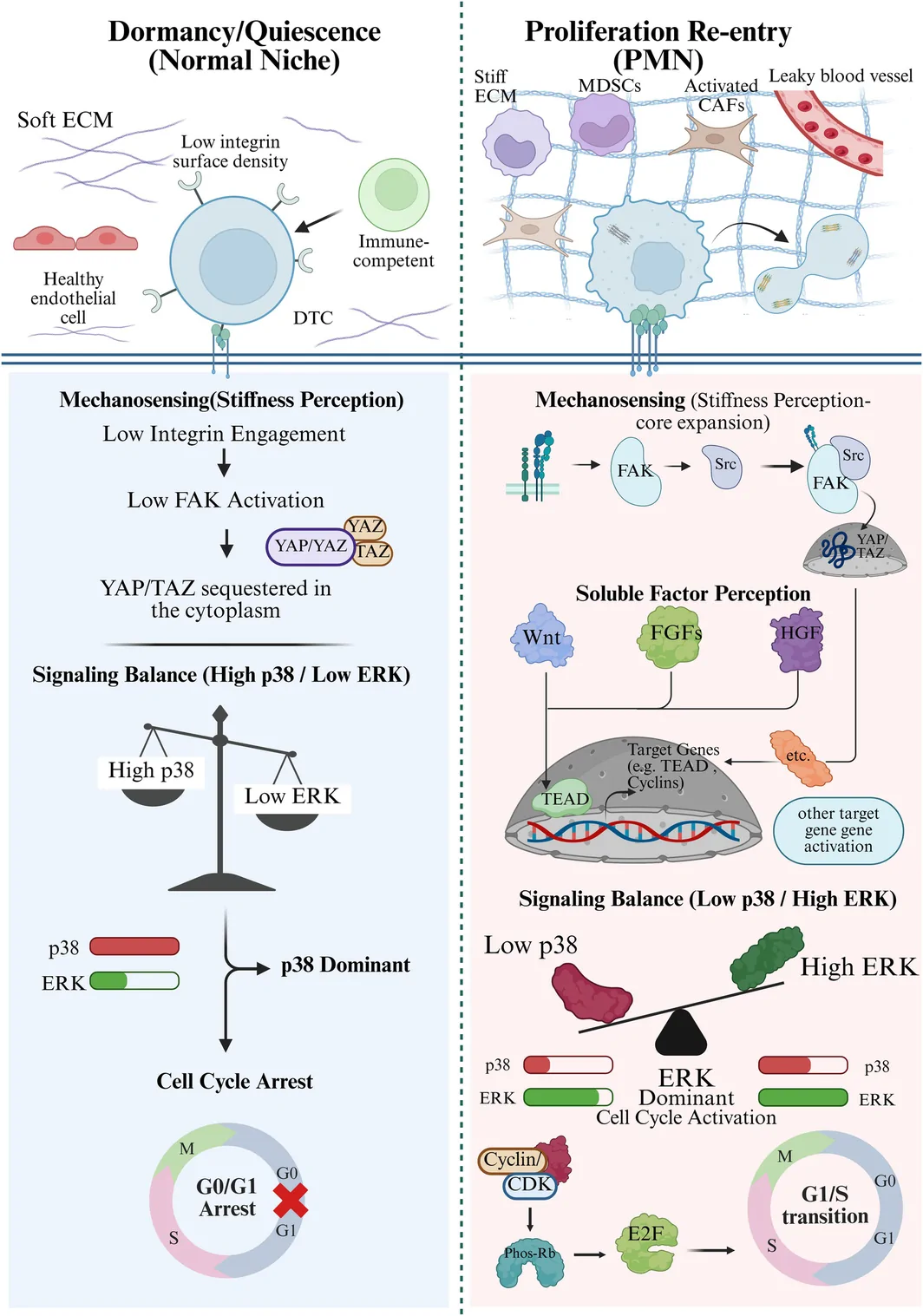
Comparison of DTC dormancy and reactivation mechanisms. Left panel: Dormancy/quiescence in the normal niche features soft extracellular matrix, low integrin surface density and an immune‐competent environment, with cytoplasmic retention of YAP/TAZ, a dominant p38 mitogen‐activated protein kinase activity and G0/G1 cell cycle arrest. Right panel: Proliferation re‐entry in the pre‐metastatic niche is driven by stiff extracellular matrix, activated cancer‐associated fibroblasts, myeloid‐derived suppressor cells and leaky blood vessels, leading to nuclear YAP/TAZ translocation, a shift to high ERK/low p38 signalling, Rb phosphorylation and activation of cell cycle target genes. Created in BioRender. Xiao, H. (2026) https://BioRender.com/z0o1w56.

### 4.1 The Sanctuary of Dormancy

Upon arriving at the pre‐metastatic niche, the fate of disseminated tumour cells is dictated not only by their intrinsic genetic and epigenetic traits but also by the precise coordination of complex biomechanical signals and biochemical stimuli from the pre‐metastatic microenvironment [90, 92, 95]. Far from being a passive failure of proliferation caused by environmental maladaptation, DTC dormancy represents a highly coordinated and reversible state of cellular quiescence that enables tumour cells to withstand metabolic stress and immune surveillance in a new environment [91, 96, 97]. During this process, the cell cycle transitions of disseminated tumour cells are instructively regulated by microenvironmental cues [98]. For example, within the bone marrow niche, NG2+/Nestin+ mesenchymal stem cells trigger downstream signalling pathways in disseminated tumour cells by secreting TGF‐β2 and BMP7 [99]. These niche‐derived factors induce a characteristically high ratio of p38 mitogen‐activated protein kinase activity relative to low extracellular signal‐regulated kinase activity in disseminated tumour cells [90, 100]. This elevated p38 to ERK ratio is widely recognized as the core signalling hallmark of dormancy, driving the upregulation of cell cycle inhibitors to enforce G0/G1 phase arrest [101, 102].

In recent years, NR2F1 has been identified as a critical molecular sentinel that integrates environmental cues with the dormancy program [102]. Robust expression of NR2F1 ensures the long‐term survival of disseminated tumour cells in distant tissues by repressing SOX9 expression and activating stemness programs [102]. Concurrently, the role of epithelial–mesenchymal transition in dormancy has been redefined. While traditionally viewed solely as a facilitator of invasion, EMT‐associated transcription factors have been found to directly mediate cell cycle arrest in cooperation with NR2F1 following cell extravasation [103]. This EMT‐induced quiescence confers profound stress tolerance upon disseminated tumour cells, enabling them to evade conventional cytotoxic therapies that target actively proliferating cells [100, 104].

Furthermore, the pre‐metastatic niche serves as an immunosuppressive sanctuary that allows dormant disseminated tumour cells to escape systemic immune surveillance [20, 48]. Within this niche, the enrichment of immunosuppressive cells such as myeloid‐derived suppressor cells and regulatory T cells creates physical and chemical barriers that significantly reduce the likelihood of effector T cells encountering rare disseminated tumour cells [73]. In addition, dormant disseminated tumour cells frequently downregulate major histocompatibility complex class I molecules to achieve immune stealth, thereby evading antigen‐specific killing by T lymphocytes [105, 106]. At the same time, epigenetic modifications, such as increased inhibitory H3K27me3 marks, further solidify this dormant phenotype, transforming transient cell cycle arrest into a stable cellular memory [107]. This epigenetic stabilization enables long‐term latency spanning years or even decades before the formation of clinically detectable macrometastases [98, 99].

### 4.2 Molecular Switches for Reactivation

The dormant state of disseminated tumour cells is not a permanent endpoint but rather a dynamic arrest that is highly reversible depending on microenvironmental conditions [98, 100]. Their re‐entry into the cell cycle fundamentally depends on the precise coupling between exogenous stimuli from the evolving pre‐metastatic niche and endogenous molecular switches within the disseminated tumour cells [99, 108]. Because disseminated cancer cells often harbour defects in key cell cycle checkpoints, such as p53 and retinoblastoma protein, along with high genomic instability, they exhibit extreme sensitivity to mitogenic signals in the microenvironment that exceed a certain threshold [90, 101, 109, 110].

As the pre‐metastatic niche progressively matures, extensive collagen crosslinking mediated by cancer‐associated fibroblasts significantly increases local extracellular matrix stiffness [55]. This mechanical alteration serves as a critical switch to awaken disseminated tumour cells [80]. Matrix stiffening triggers integrin clustering on the surface of disseminated tumour cells, which potently activates the focal adhesion kinase and Src signalling cascades [111]. This mechanotransduction process subsequently induces the dephosphorylation and nuclear translocation of YAP and TAZ, which function as downstream effectors of the Hippo pathway [112, 113]. Upon entering the nucleus, YAP and TAZ act as crucial transcriptional coactivators that directly drive the transcription of mitogenic target genes such as MYC and CCND1, thereby propelling disseminated tumour cells across the G1/S restriction point [114].

Beyond physical biomechanical cues, acute and chronic inflammatory factors enriched within the pre‐metastatic niche constitute another class of critical reactivation switches [115]. For instance, under systemic inflammation or chemotherapy‐induced stress, neutrophils recruited to the pre‐metastatic niche release neutrophil extracellular traps [116]. The neutrophil elastase and matrix metalloproteinase 9 delivered by these traps not only degrade the stroma but also specifically cleave laminin. The resulting activation fragments bind to and stimulate α3β1 integrin receptors on the surface of disseminated tumour cells, thereby activating the downstream FAK/ERK pathway and directly initiating the proliferative program. Furthermore, dynamically changing TGF‐β cascades and Wnt/β‐catenin pathways within the microenvironment can breach the threshold required for dormancy maintenance.

During the execution phase, all upstream mechanical and biochemical signals ultimately converge on the derepression of the core cell cycle machinery [117, 118]. Driven by sustained stimulation from pre‐metastatic niche maturation signals, the endogenously upregulated cyclin‐dependent kinase inhibitors in disseminated tumour cells undergo accelerated degradation via the ubiquitin‐proteasome pathway. Concurrently, epigenetic remodelling, such as the erasure of the inhibitory histone modification H3K27me3, downregulates the expression of dormancy‐related transcription factors including NR2F1 [119]. These synergistic events allow cyclin‐dependent kinase 4/6‐cyclin D complexes to reassemble and become activated. This activation leads to the phosphorylation of Rb and the subsequent release of E2F transcription factors, fully restarting the DNA replication program [120].

Based on this dynamic cascade, the transition of disseminated tumour cells into a proliferative state can be viewed as the ultimate functional readout of pre‐metastatic niche maturity [48]. The resurgence of disseminated tumour cells is not an isolated spontaneous event but rather a qualitative transition that occurs once the establishment of immunosuppressive networks, enrichment of metabolic substrates and matrix mechanical remodelling within the pre‐metastatic niche accumulate to a critical threshold [115]. The key molecular switches and sensing mechanisms that dictate DTC fate by balancing dormancy maintenance and proliferative re‐entry are summarized in Table 3.

**TABLE 3:** Molecular switches and signalling pathways regulating the transition between DTC dormancy and proliferative re‐entry.

Molecular category	Dormancy‐promoting mechanisms	Proliferation‐promoting mechanisms	References
Transcription factors	NR2F1: induces G0/G1 cell cycle arrest and maintains stemness; represses SOX9 transcription.	SOX9: promotes epithelial–mesenchymal transition and cell cycle re‐entry.	[102]
Signal transduction	p38 MAPK: high p38β activity relative to ERK promotes quiescence and survival.	High ERK/MAPK activity drives cell cycle progression and proliferation.	[90]
Microenvironment sensors	Hippo pathway: activation of LATS1/2 leads to cytoplasmic retention of YAP/TAZ.	YAP/TAZ: nuclear translocation in response to increased extracellular matrix stiffness (mechanotransduction).	[121]
TGF‐β superfamily	TGF‐β2: activates p38 to induce dormancy in the bone marrow microenvironment.	TGF‐β1: promotes SMAD4‐dependent outgrowth and metastatic colonization.	[122]
Epigenetic regulators	EZH2 (Low): low levels allow expression of dormancy genes.	EZH2: deposits H3K27me3 marks to repress tumour suppressors (RUNX3).	[123]

## 5 Multidimensional Drivers: Metabolism and Epigenetics in Pre‐Metastatic Niche

During multicellular reprogramming, metabolic resetting and epigenetic modifications together form the underlying molecular logic that governs the formation and maintenance of the pre‐metastatic niche. These two processes are mutually coupled, allowing the niche to achieve a relatively stable and sustainable pathological state.

### 5.1 Metabolic Reprogramming in the Niche

Metabolic remodelling within the pre‐metastatic niche provides the essential energetic and biosynthetic substrates for phenotypic transitions of resident cells and the subsequent colonization of disseminated tumour cells. Accumulating evidence indicates that systemic alterations in glucose, lipid and amino acid metabolism cooperatively orchestrate pre‐metastatic niche activation [124].

Regarding glucose metabolism, hepatocellular carcinoma‐derived EVs are internalized by lung fibroblasts, significantly suppressing their glucose uptake and utilization. This remote suppression establishes a relatively glucose‐enriched microenvironment in the pulmonary pre‐metastatic niche, reserving critical carbon sources for rapid DTC proliferation [125]. Notably, pre‐metastatic niches in different metastatic target organs display pronounced metabolic heterogeneity. While the pulmonary pre‐metastatic niche is often characterized by glucose enrichment, the hepatic pre‐metastatic niche relies more heavily on lipid metabolism to shape its pro‐inflammatory microenvironment [126]. In this process, alterations in extracellular matrix stiffness can induce metabolic adaptations in tumour cells through mechanotransduction and modulate exosomal microRNA expression profiles, thereby remotely remodelling the metabolic landscape of target organs [127, 128]. Furthermore, exogenous dietary factors can systemically modulate microenvironmental metabolism. For instance, a high‐fat diet activates platelets and endothelial cells, accelerating fibronectin deposition and exacerbating vascular leakage [129]. Conversely, caloric restriction suppresses pre‐metastatic niche formation to some extent by improving systemic metabolic homeostasis and modulating immune cell infiltration patterns in the distal microenvironment [130, 131].

With respect to lipid metabolism, the hypoxic response mediated by hypoxia‐inducible factor 1‐alpha drives lipid droplet accumulation in macrophages. These macrophages subsequently transfer lipids to cytotoxic T lymphocytes via EVs, inducing immunometabolic exhaustion while concurrently enhancing the glycolytic capacity of tumour cells, thereby establishing a pro‐metastatic environment characterized by nutrient enrichment and immunosuppression [126]. Lipid metabolic reprogramming in cancer‐associated fibroblasts also serves as a critical driver of pre‐metastatic niche development. Studies have shown that colorectal cancer cell‐derived exosomal HSPC111 promotes the phosphorylation of ATP citrate lyase, which increases acetyl‐coenzyme A synthesis in cancer‐associated fibroblasts. This metabolic shift stimulates the secretion of pro‐inflammatory cytokines such as CXCL5, ultimately constructing a pro‐metastatic microenvironment in the liver [56]. Additionally, EVs released by *Fusobacterium nucleatum* ‐infected colorectal cancer cells can deliver lipid metabolism‐associated molecules and activate immunosuppressive signalling pathways to promote pre‐metastatic niche formation [132, 133]. In pancreatic ductal adenocarcinoma, the metabolic reprogramming of cancer‐associated fibroblasts can be mediated by tumour‐derived extracellular vesicles through a SMAD4‐dependent pathway, further underscoring the vital role of lipid metabolism in shaping the pre‐metastatic niche [134].

At the amino acid level, tumour‐derived EVs can reprogram the metabolic patterns of pre‐metastatic niche‐resident cells by delivering specific miRNAs [135]. This reprogramming not only restricts their glucose utilization but also redirects the metabolic flux of amino acids, particularly glutamine, thereby reserving indispensable nitrogen sources and carbon skeletons for DTC proliferation [135]. Broader systemic analyses suggest that metastatic tumour cells typically employ dual metabolic strategies within the pre‐metastatic niche: actively shaping the metabolic characteristics of the microenvironment while passively adapting to local tissue‐specific metabolic constraints. The synergy between these two strategies facilitates the cultivation of a fertile soil conducive to colonization [124].

In recent years, the coupling mechanisms between metabolism and immunity have attracted significant attention. The activation of glycolysis in neutrophils within the pre‐metastatic niche not only remodels the local tumour microenvironment but also induces immunosuppression through the release of specific metabolites [136]. Moreover, metabolic crosstalk between tumour cells and platelets can modulate circulating metabolite levels, thereby influencing pre‐metastatic niche formation and the colonization efficiency of disseminated tumour cells in distant organs [129].

### 5.2 Organ‐Specific Signatures of the ‘Educated Soil’

To comprehensively capture the ‘educated soil’ concept, it is essential to recognize that the pre‐metastatic niche exhibits profound organotropism. Primary tumours dictate the preferential colonization of high‐incidence metastatic organs, namely the lung, liver, bone and brain, by orchestrating distinct patterns of stromal remodelling, immune recruitment and metabolic rewiring tailored to the intrinsic physiology of each target site. A systematic comparison of these four major niches reveals the precise, organ‐specific logic of remote tumour education:

*Lung* (*pulmonary niche*): The pulmonary pre‐metastatic niche is structurally defined by robust vascular hyperpermeability and fibrotic remodelling [37, 137]. Tumour‐derived EVs (e.g., enriched in miR‐105 or VEGF) disrupt tight junctions in endothelial cells, while resident lung fibroblasts are activated to deposit a stiffened ECM rich in collagen and fibronectin [138]. Immunologically, the lung niche is dominated by a massive recruitment of neutrophils and granulocytic myeloid‐derived suppressor cells. Notably, recruited neutrophils can deploy neutrophil extracellular traps, providing local scaffolds that capture disseminating cells and release proteases to awaken them from dormancy [46, 139]. Metabolically, the lung PMN is a glucose‐enriched environment; primary tumours remotely suppress glucose uptake in resident fibroblasts, thereby sparing abundant glucose substrates to fuel the rapid glycolytic expansion of colonizing cancer cells [125, 140, 141].

*Liver* (*hepatic niche*): In stark contrast, the hepatic pre‐metastatic niche is fundamentally lipid‐focused. Stromal remodelling is driven by the activation of HSCs into myofibroblasts, leading to dense localized fibronectin deposition [142, 143]. The immune landscape is highly immunosuppressive, characterized by the influx of monocytic MDSCs and the reprogramming of resident Kupffer cells into lipid‐laden, pro‐tumourigenic macrophages. This local microenvironment relies heavily on lipid metabolic rewiring [126, 144, 145, 146]. For instance, exosomal factors (such as HSPC111) reprogram lipid metabolism in hepatic cancer‐associated fibroblasts to stimulate the secretion of pro‐inflammatory cytokines like CXCL5 [56]. Concurrently, lipid‐laden macrophages facilitate T‐cell exhaustion, establishing an immune‐privileged and lipid‐enriched soil ideal for hepatic colonization [126].

*Bone* (*osteogenic*/*osteolytic niche*): The bone pre‐metastatic niche is sculpted by the profound dysregulation of bone homeostasis, specifically the early hyperactivation of osteoclasts prior to tumour arrival. This stromal destruction releases matrix‐bound growth factors (such as TGF‐β) and calcium, creating a fertile physical scaffold. Immunologically, the bone marrow, a natural reservoir for immune progenitors, is skewed toward an immature, immunosuppressive myeloid phenotype, populated by M2‐like macrophages and regulatory T cells (Tregs) [147, 148]. Metabolically, the bone PMN is inherently hypoxic and acidic. To overcome this extreme metabolic stress, bone marrow mesenchymal stem cells engage in metabolic crosstalk with arriving tumour cells, such as transferring functional mitochondria via tunnelling nanotubes or extracellular vesicles, thereby upgrading the oxidative phosphorylation capacity and metabolic adaptability of the disseminated seeds [149, 150, 151].

*Brain* (*cerebral niche*): The critical initiating event in the brain PMN is the pre‐metastatic disruption of the highly regulated blood–brain barrier. Tumour‐secreted factors downregulate endothelial tight junction proteins, enabling targeted vascular leakiness [37, 138]. Concurrently, brain‐specific stromal remodelling involves reactive astrogliosis and the activation of resident microglia [152]. While the blood–brain barrier inherently restricts peripheral immune infiltration, its focal pre‐metastatic breakdown allows specific bone marrow‐derived macrophages to enter and synergize with reprogrammed, immunosuppressive microglia [153]. Metabolically, because the central nervous system is highly dependent on glucose and specific amino acids, arriving tumour cells rely on reactive astrocytes. These astrocytes are metabolically rewired to supply the colonizing tumour cells with critical nutrients, including glutamine and branched‐chain amino acids, fulfilling their high biosynthetic demands in a nutrient‐restricted environment [154, 155, 156].

By systematically mapping these organ‐specific signatures, it becomes evident that the pre‐metastatic niche is not a uniform biological response, but rather a highly customized, target‐specific ecological remodelling process. Recognizing these distinct stromal, immune and metabolic vulnerabilities across the lung, liver, bone and brain is paramount for developing precision interceptive therapies.

### 5.3 Epigenetic Modification of the Cellular State

Epigenetic modifications translate environmental and metabolic cues into stable cellular phenotypic memories. In bone metastasis models, amplification of the histone methyltransferase ASH1L gene synergizes with hypoxia‐inducible factor 1‐alpha to induce insulin‐like growth factor 2 secretion by tumour cells. This process alters histone methylation patterns, driving the differentiation of monocytes within the bone microenvironment into lipid‐associated tumour‐associated macrophages [157]. Concurrently, the H3K4 methyltransferase SMYD3 enhances H3K4me3 modifications at the promoter regions of EMT‐associated transcription factors, thereby promoting an invasive phenotype in colorectal cancer cells [158].

Non‐coding RNAs, such as miR‐934, can suppress target gene expression via post‐transcriptional regulation and activate the PI3K/AKT signalling pathway, thereby sustaining the M2‐like polarization of macrophages. Furthermore, various ncRNAs, including microRNAs and long non‐coding RNAs delivered by extracellular vesicles, modulate target gene expression at the post‐transcriptional level [159]. These molecules play critical roles in driving immunosuppression, angiogenesis and extracellular matrix remodelling within the pre‐metastatic niche. Metabolic intermediates, such as acetyl‐coenzyme A, directly serve as substrates for epigenetic modifications. They form a feedback regulatory network with epigenetic enzymes, which sustains the collaborative multicellular state. Recent studies further emphasize that metabolic fluctuations within the tumour microenvironment can alter the activity of epigenetic enzymes, subsequently remodelling the phenotypes and functions of immune cells [160, 161].

Research into the precancerous niche has expanded the temporal framework of this concept, indicating that epigenetic remodelling may initiate during the earliest stages of tumourigenesis. As cancer‐associated fibroblasts are among the most abundant stromal cells in the pre‐metastatic niche, their epigenetic state directly impacts metastatic potential [162]. Recent reviews have systematically outlined the diverse origins of cancer‐associated fibroblasts, their multidimensional roles in pre‐metastatic niche formation and potential therapeutic strategies aimed at disrupting the pre‐metastatic niche by targeting these cells. Additionally, autophagy, a cellular stress response regulated by epigenetic mechanisms, enables tumour cells to survive under metabolic stress and enter a dormant state [163]. To accurately elucidate the complex multicellular interactions and spatiotemporal dynamics within the pre‐metastatic niche, a range of cutting‐edge single‐cell and spatial omics technologies have been widely employed; their key features and research advantages are summarized in Table 4.

**TABLE 4:** Emerging high‐resolution technologies and computational frameworks for deciphering the spatiotemporal heterogeneity of the pre‐metastatic niche.

Technology/method	Core technical features and advantages	Evidence level	Major limitations	Translational readiness	References
Single‐cell RNA sequencing (scRNA‐seq)	Captures transcriptomes of isolated single cells; resolves rare subclusters and cellular heterogeneity	In vitro, mouse model, human specimen	Dissociation bias, loss of spatial topology, artificial stress gene induction, limited causal inference.	Preclinical validation	[164]
Spatial transcriptomics (ST)	Profiles gene expression in situ while preserving tissue architecture and spatial ligand‐receptor networks.	Mouse model, human specimen, clinical cohort	Spatial resolution trade‐offs, cell‐type annotation uncertainty, high cost, static snapshot.	Preclinical validation	[165]
Spatial multi‐omics	Integrates transcriptomic, proteomic, epigenetic, or metabolomic layers on identical/adjacent tissue sections.	Mouse model, human specimen	Small sample size, high cost, cross‐platform alignment artefacts, algorithm dependence.	Exploratory	[166]
Single‐cell/spatial metabolomics	Mass spectrometry imaging captures in situ spatial gradients of lipids and energy metabolites.	In vitro, mouse model, human specimen	Lower sensitivity for low‐abundance metabolites, high cost, limited causal inference.	Exploratory	[167]
scATAC‐seq and spatial ATAC‐seq	Profiles chromatin accessibility dynamics to reveal pre‐transcriptional chromatin remodelling.	In vitro, mouse model, human specimen	High data sparsity/dropouts, small sample size, algorithm dependence, cell‐type annotation uncertainty.	Exploratory	[168, 169]
Computational frameworks and AI integration	Graph models and machine learning integrate multi‐modal spatial data for spatial risk scoring.	Mouse model, human specimen, clinical cohort	Algorithm dependence, risk of overfitting on small cohorts, ‘black‐box’ predictions, limited causal inference.	Exploratory to Clinically Applicable	[170]

## 6 Emerging Technologies for Pre‐Metastatic Niche Analysis

To accurately elucidate the complex and dynamic multicellular interactions within pre‐metastatic niches, the research paradigm is shifting from traditional bulk sequencing to high‐resolution single‐cell analysis and three‐dimensional spatial omics technologies. To precisely characterize the intricate multicellular coordination and spatiotemporal evolution within pre‐metastatic niches, a range of cutting‐edge single‐cell, spatial omics and real‐time imaging techniques is being widely employed (Figure 4).

**FIGURE 4:**
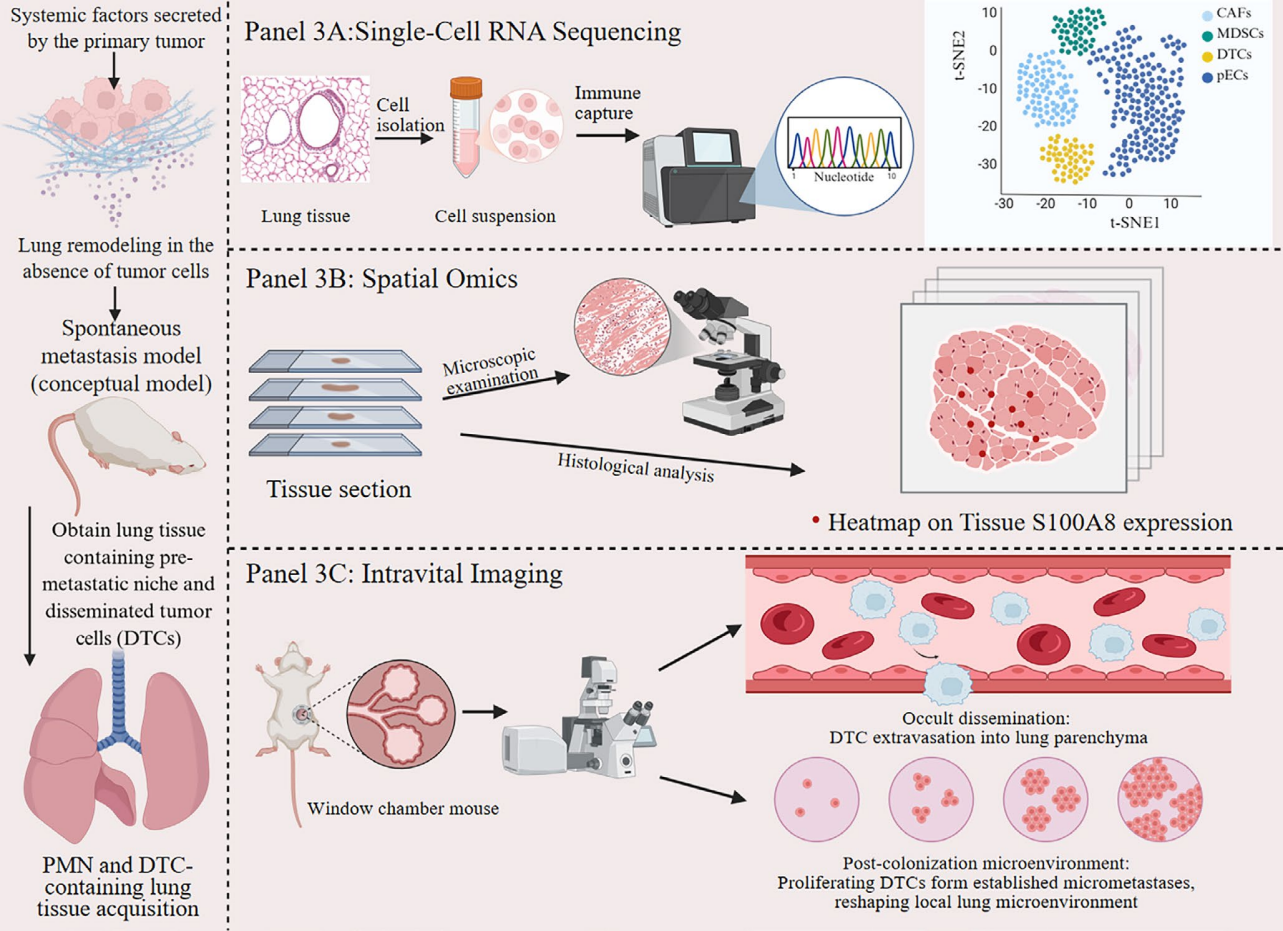
Overview of cutting‐edge technologies for pre‐metastatic niche analysis. (A) Single‐cell RNA sequencing reveals cellular heterogeneity and rare subpopulations within the pre‐metastatic niche. (B) Spatial omics preserves tissue architecture to map molecular features (S100A8) and ligand‐receptor networks in situ. (C) Intravital imaging enables real‐time tracking of dynamic processes such as tumour cell extravasation and proliferation in living pre‐metastatic niche models. Created in BioRender. Xiao, H. (2026), https://BioRender.com/z0o1w56.

### 6.1 Single‐Cell and Spatial Transcriptomics

Single‐cell RNA sequencing reveals the profound cellular heterogeneity within the pre‐metastatic niche at high resolution. It enables precise identification of cancer‐associated fibroblasts with distinct pro‐metastatic functions, as well as rare immune cell subpopulations. For instance, application of single‐cell RNA sequencing in osteosarcoma has elucidated complex intercellular communication networks between malignant and stromal cells, establishing a robust technical paradigm for deciphering cellular heterogeneity within the pre‐metastatic niche [171, 172].

Furthermore, single‐cell RNA sequencing provides a high‐resolution view of the tumour ecosystem within distant metastatic niches. In a recent multicentre study on breast cancer brain metastases, researchers used single‐cell RNA sequencing together with Monocle2 and CellChat algorithms to map the metastatic microenvironment, lineage trajectories and intricate cell–cell communication networks. This analysis revealed profound phenotypic heterogeneity across major cell lineages and identified interleukin enhancer‐binding factor 2 as a key metastasis‐driving gene. Crucially, functional validations showed that deficiency of this identified factor significantly halted breast cancer brain metastasis progression, underscoring its clinical potential as a biomarker and therapeutic target for metastatic interception [173].

Building on these single‐cell insights, spatial transcriptomics preserves in situ tissue architecture, allowing researchers to map ligand–receptor interaction networks across precise spatial coordinates. For example, stClinic, a dynamic graph model, integrates spatial multimodal data to systematically identify malignant niches characterized by enrichment of tumour‐associated macrophages, as well as B cell‐rich niches that predict a favourable prognosis. Ultimately, such topological analyses provide a critical spatial framework for pinpointing key regions of pre‐metastatic niche formation and identifying niche‐specific therapeutic targets [82].

Crucially, the translational value of spatial multi‐omics extends far beyond deciphering basic cell–cell interactions; it provides a powerful framework for clinical risk stratification and precision oncology. Bulk and single‐cell sequencing often lose the crucial topological context required to define a functional pre‐metastatic niche. By preserving architecture, spatial transcriptomics enables the identification of niche‐specific immune signatures, such as the focal spatial aggregation of M2‐like TAMs, the physical exclusion of CD8+ effector T cells by fibrotic CAF barriers, or the loss of protective tertiary lymphoid structure‐like B‐cell niches [174, 175]. Quantifying these spatially constrained multicellular hubs allows for the extraction of high‐dimensional “spatial risk scores.” When integrated with digital pathology and machine learning, these spatial immune signatures can identify patients at high risk of rapid PMN priming and early metastatic recurrence before overt macrometastases are clinically detectable [176, 177, 178]. Consequently, bridging spatial omics with clinical diagnostics offers a window for pre‐emptive patient stratification, guiding early implementation of tailored microenvironment‐targeted or adjuvant immunotherapies.

### 6.2 Organ‐On‐a‐Chip and Organoid Models

Given the limitations of traditional animal models in real‐time monitoring and human‐specific replication, microfluidic organ‐on‐a‐chip and three‐dimensional organoid technologies have emerged as vital in vitro engineering platforms for pre‐metastatic niche research. Using multi‐chamber architectures and microfluidic perfusion, organ‐on‐a‐chip devices can co‐culture tumour, endothelial and immune cells. This setup effectively replicates key pre‐metastatic niche dynamics, including vascular hyperpermeability, recruitment of circulating factors and extracellular matrix deposition [179, 180]. To date, these platforms have successfully modelled solid malignancies such as pancreatic, colorectal and breast cancers by simulating tripartite tumour–stroma–immune interactions, thereby offering a highly controllable system to dissect niche‐specific intercellular communication [181]. Furthermore, integrating these biomimetic models with CRISPR‐Cas9 gene editing accelerates functional validation of specific metabolic enzymes or epigenetic regulators involved in cross‐organ reprogramming, establishing a robust foundation for screening lead compounds designed to disrupt pre‐metastatic niche networks [182, 183].

### 6.3 Methodological Bottlenecks, Analytical Challenges and Biological Caveats

While single‐cell, spatial‐omics and intravital imaging platforms have revolutionized our capacity to decode the PMN, their application is accompanied by severe technical limitations, analytical hurdles and conceptual caveats that must be critically acknowledged:

*Tissue‐dissociation bias and loss of rare populations*: Single‐cell RNA sequencing fundamentally relies on enzymatic and mechanical tissue dissociation, which preferentially destroys fragile stromal, endothelial or matrix‐embedded cell types while inducing artificial stress‐response gene expression signatures [184, 185]. Crucially, this dissociation bias frequently leads to the loss or underrepresentation of ultra‐rare, matrix‐anchored PMN‐initiating populations (e.g., early recruited specific myeloid subsets or rare niche‐initiating stromal clones) [186, 187, 188].

*Spatial resolution limits and cell‐type annotation ambiguity*: Spatial transcriptomic technologies face an inherent trade‐off between transcriptomic depth and spatial resolution. Sequencing‐based platforms (e.g., 10× Visium) capture multi‐cellular spots (55 μm diameter) rather than true single‐cell resolution, introducing significant cell‐type deconvolution ambiguity and annotation uncertainty. Conversely, imaging‐based platforms offer subcellular resolution but remain restricted to targeted gene panels.

*Batch effects, sample‐size constraints and cross‐platform integration*: High experimental costs frequently restrict study designs to small animal cohort sizes, compromising statistical power [189, 190]. Furthermore, technical batch effects across experimental runs pose major analytical challenges, while integrating multi‐modal spatial datasets (e.g., pairing spatial transcriptomics with spatial metabolomics or ATAC‐seq on adjacent tissue sections) requires complex computational alignment algorithms that risk introducing spatial registration artefacts [191].

*Biological ambiguity: true pre‐metastatic priming versus occult DTC microenvironments*: A paramount biological challenge in PMN research is definitively distinguishing a *truly pre‐metastatic niche* (primed solely by primary‐tumour‐derived systemic signals) from a microenvironment harbouring undetectable, occult DTCs. Bulk or coarse‐resolution spatial analyses risk misattributing local microenvironmental responses triggered by microscopic, occult DTCs to true remote primary‐tumour‐driven PMN education.

*Descriptive associations versus causal biological mechanisms*: High‐throughput multi‐omics profiling predominantly yields descriptive, correlative interaction maps and ligand–receptor prediction networks. These high‐dimensional associations lack functional directionality; high‐throughput correlation does not inherently equal biological causation. Rigorous functional perturbation assays, such as CRISPR‐mediated cell‐type‐specific gene knockout in organoid/chip models or conditional mouse knockouts, are strictly required to establish causality [192, 193, 194, 195].

Consequently, overcoming these bottlenecks requires moving beyond single‐platform descriptive studies toward integrative, multi‐modal workflows that combine high‐resolution spatial discovery with rigorous functional perturbation and standardized bioinformatic validation.

## 7 Therapeutic Vulnerabilities: Toward Microenvironment Normalization

Mechanistic studies indicate that pre‐metastatic niche formation relies on systemic signalling between the primary tumour and distant organs, multicellular reprogramming and coordinated metabolic‐epigenetic regulation [1, 9, 97]. This dynamic process exposes multiple actionable therapeutic vulnerabilities. Unlike traditional strategies that directly target tumour cells, pre‐metastatic niche‐directed therapies aim to block distant soil remodelling, restore immune surveillance and exploit the cell cycle transition window of disseminated tumour cells for precise intervention [6, 196, 197, 198].

Consequently, the core therapeutic paradigm is shifting from simple killing to normalization, offering novel strategies for the early prevention and intervention of metastasis [199, 200]. Interventions against pre‐metastatic niche formation and DTC proliferation can be categorized as multi‐target niche strategies, encompassing the blockade of primary tumour signals, microenvironmental reprogramming and the modulation of DTC dormancy [90, 201, 202]. Notably, the drug development pipeline for these approaches has successfully advanced from early discovery to clinical trials (Figure 5).

**FIGURE 5:**
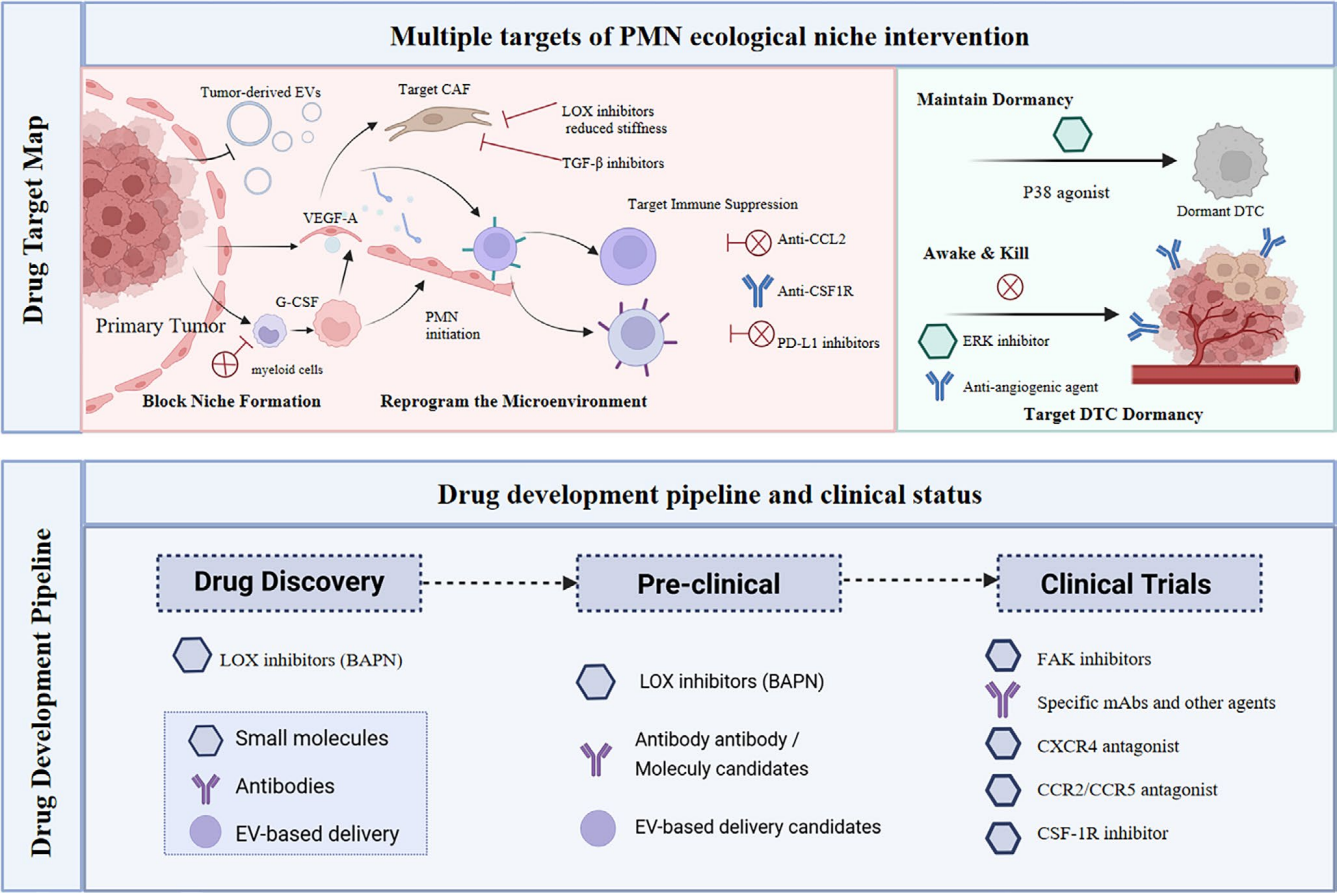
Overview of therapeutic vulnerabilities and drug discovery in the pre‐metastatic niche. Key intervention points include blocking systemic signals from the primary tumour, reprogramming the immunosuppressive and fibrotic microenvironment and either maintaining DTC dormancy or inducing awaken and kill strategies. The accompanying pipeline illustrates the progress of representative agents (LOX inhibitors, focal adhesion kinase inhibitors, p38 agonists) from drug discovery through preclinical and clinical phases. Created in BioRender. Xiao, H. (2026) https://BioRender.com/z0o1w56.

### 7.1 Blocking Long‐Distance Communication

The initiation of the pre‐metastatic niche depends on systemic signals released by the primary tumour, which drive the proliferation and functional transformation of distant resident cells [203]. Extracellular vesicles, including EVs, serve as major vehicles for this long‐range communication. They transport proteins, nucleic acids and lipids through the circulation to deliver biological information to specific target organs [11, 204]. Therefore, blocking this remote tumour communication represents a key early strategy for intercepting the metastatic cascade.

#### 7.1.1 Pharmacological Inhibition of EV Biosynthesis and Secretion

Intercepting exosomal communication directly involves suppressing vesicle biogenesis and release within primary tumour cells [205]. EV formation proceeds through the endosomal pathway, where multivesicular bodies fuse with the plasma membrane to release their cargo. Rab family small GTPases, particularly Rab27a and Rab27b, play crucial roles in regulating multivesicular body transport and docking [206]. Downregulating Rab27a via small interfering RNA or small‐molecule inhibitors reduces tumour‐derived EV secretion, thereby attenuating the remote education of target cells and suppressing neutrophil recruitment as well as inflammatory niche establishment [63, 207].

Neutral sphingomyelinase 2 is another essential enzyme that regulates EV budding [208]. The small‐molecule inhibitor GW4869 suppresses sphingomyelin hydrolysis, reducing lipid raft formation on multivesicular body membranes and thereby disrupting pro‐metastatic cargo loading and vesicle release [209]. In small cell lung cancer and breast cancer models, GW4869 impairs pre‐metastatic niche formation and enhances systemic immune surveillance by lowering circulating levels of exosomal programmed death‐ligand 1 [210, 211, 212].

#### 7.1.2 Neutralization and Clearance of Circulating Pro‐Metastatic Factors

Beyond inhibiting release, neutralizing or clearing circulating pro‐metastatic extracellular vesicles and soluble factors represents another important therapeutic approach [213]. Tumour‐derived EVs exhibit distinct organ tropism dictated by their surface integrin expression profiles. For example, EVs expressing α6β4 preferentially home to the lungs, whereas those bearing αvβ5 target the liver [11]. Developing monoclonal antibodies or competitive ligands against these integrins can block EV binding to resident cells, thereby preventing the pre‐metastatic tilling of distant organs [11, 20].

In addition, hemofiltration technologies using microfluidic devices or specific affinity membranes are being explored to physically deplete circulating tumour‐associated extracellular vesicles, thus reducing their exposure and accumulation in distant organs [213, 214].

While preclinical strategies aimed at intercepting PMN priming, such as pharmacological blockade of EV biogenesis (e.g., GW4869) or chemokine receptor neutralization, have yielded remarkable anti‐metastatic efficacy in syngeneic mouse models of breast, renal and colorectal cancers, their therapeutic vulnerabilities are highly context‐dependent. The degree of PMN inhibition achieved by targeting specific pathways varies significantly depending on primary tumour histology, host immune status and the intrinsic biomechanical properties of the target metastatic organ. Therefore, therapeutic interventions directed against the PMN should be framed as context‐specific strategies tailored to particular tumour‐organ combinations rather than generalized anti‐metastatic solutions for all solid tumours.

### 7.2 Reshaping the Immune Microenvironment

A core hallmark of the pre‐metastatic niche is the establishment of an immunosuppressive microenvironment that evades immune surveillance [20, 215]. Within this niche, the massive expansion of myeloid‐derived suppressor cells and the M2‐like polarization of macrophages are critical events that support the survival and colonization of disseminated tumour cells [216]. Eliminating, blocking, or functionally reprogramming these immunosuppressive components are vital strategies for restoring anti‐tumour immunity in target organs [217, 218].

#### 7.2.1 Clearance and Recruitment Inhibition: Dismantling the MDSC Defence Network

Myeloid‐derived suppressor cells are immature myeloid cells with potent immunosuppressive functions. Driven by tumour‐derived factors such as vascular endothelial growth factor, interleukin‐6 and tumour necrosis factor‐alpha, they are mobilized from the bone marrow and accumulate in distant organs [73, 219]. These cells inhibit T‐cell proliferation and activation by releasing reactive oxygen species, arginase‐1 and inducible nitric oxide synthase [220, 221]. Therapeutic strategies targeting MDSCs mainly include inducing their differentiation, blocking their recruitment and suppressing their function. Drugs such as all‐trans retinoic acid induce MDSC differentiation into mature granulocytes or monocytes with reduced immunosuppressive capacity [222, 223]. Small‐molecule CCR2 antagonists block the CCL2‐CCR2 axis to reduce monocytic MDSC accumulation in organs such as the lungs and liver [224]. Additionally, inhibitors of inducible nitric oxide synthase or arginase can partially restore T‐cell effector functions [225].

#### 7.2.2 Reprogramming Macrophage Polarization: From Accomplices to Sentinels

During pre‐metastatic niche formation, resident macrophages are frequently reprogrammed by primary tumour signals into a pro‐tumourigenic M2‐like phenotype [142, 226]. These M2‐like macrophages secrete transforming growth factor‐beta, IL‐10 and vascular endothelial growth factor, promoting matrix remodelling, angiogenesis and immunosuppression to support DTC colonization [142, 226, 227].

Reprogramming macrophage polarization has emerged as an important direction for immune intervention in the pre‐metastatic niche. Studies indicate that metabolic reprogramming fundamentally determines macrophage phenotypes. For example, cholesterol metabolism modulated by the SENP1‐Sirt3 axis maintains the M2‐like state [228, 229]. Inhibiting related metabolic pathways or applying colony‐stimulating factor 1 receptor inhibitors can reduce tumour‐associated macrophage survival and promote their repolarization toward a pro‐inflammatory M1‐like state [230, 231]. Furthermore, trained immunity strategies use stimuli such as β‐glucan to epigenetically reprogram bone marrow myeloid progenitors. This approach yields differentiated macrophages with enhanced anti‐tumour activity, fundamentally altering the immune ecology of the pre‐metastatic niche at its source [232, 233].

#### 7.2.3 Synergistic Interventions: Regulatory T Cells and Immune Checkpoints

In the mid‐to‐late stages of pre‐metastatic niche formation, the accumulation of regulatory T cells further consolidates the immunosuppressive state. Tregs secrete IL‐35 and interfere with antigen presentation, rendering the pre‐metastatic niche a cold microenvironment with poor responses to immunotherapy [234, 235]. Combining myeloid‐targeted therapies with immune checkpoint inhibitors can generate synergistic effects [236, 237]. For instance, initially normalizing the vasculature to improve pre‐metastatic niche oxygenation and drug delivery, followed by administration of checkpoint inhibitors, has the potential to enhance the infiltration density and cytotoxic efficiency of effector T cells within the pre‐metastatic niche [238, 239].

A detailed comparison of interventions targeting various inhibitory immune components and matrix components within the pre‐metastatic niche, along with representative drugs and their expected therapeutic outcomes, is provided in Table 5.

**TABLE 5:** Therapeutic strategies and pharmacological agents targeting immunosuppressive and stromal components in the pre‐metastatic niche.

Target cells	Intervention measures	Representative drugs/targets	Therapeutic expectation	References
MDSCs	Mobilize to block/induce differentiation	CCR2 antagonists/ATRA	Reduce the infiltration of immunosuppressive cells	[240, 241]
TAMs (M2)	CSF‐1R inhibitors/Sirt3 axis	CSF‐1R inhibitors/Sirt3 axis	Restore the pro‐inflammatory and anti‐tumour state of the microenvironment	[242, 243]
Fibroblasts	Activation inhibition/extracellular matrix degradation	TGF‐β inhibitors/LOX inhibitors	Lower physical barriers and improve drug penetration	[244, 245]
Tregs	Exhaustion/checkpoint blocking	Anti‐CTLA‐4/CXCR4 inhibitor	Release T‐cell effector function	[246, 247]
Neutrophils	Exhaustion/checkpoint blocking	DNase I/PAD4 inhibitor	Block the colonization promotion effect induced by neutrophils	[248, 249]
NK cells	Reactivating anti‐tumour immunity/alleviating killing suppression	Anti‐NKG2A/IL‐15 agonists	Reverse immune evasion within the pre‐metastatic niche and restore early‐stage immune surveillance in distant organs	[250, 251]
Endothelial cells/vascular components	Attenuating vascular leakage/suppressing adhesion molecule expression	Anti‐VEGFR2/anti‐VLA‐4	Maintain endothelial barrier integrity and prevent the adhesion and extravasation of circulating tumour cells (CTCs)	[252, 253]
Soluble chemokines and cytokines	Neutralizing targeted antibodies/receptor antagonists	Anti‐CCL2/CXCR4 antagonists	Block the remote recruitment of bone marrow‐derived cells and suppress the pre‐metastatic inflammatory cascade in distant organs	[254, 255]

### 7.3 Modulating DTC Status: Balancing Dormancy Maintenance and Precision Activation

Once disseminated tumour cells reach the pre‐metastatic niche, their fate is tightly regulated by microenvironmental signals. The re‐entry of these cells from G0‐phase dormancy into the active cell cycle represents the critical rate‐limiting step in the progression from micrometastases to overt metastatic lesions [115, 256]. Consequently, therapeutic strategies diverge into two complementary directions: maintaining dormancy to delay or prevent metastasis, or inducing DTC proliferation followed by cell‐cycle‐specific eradication [102, 257].

#### 7.3.1 Maintaining Dormancy: Strategies to Delay Metastatic Progression

Metastatic relapse can occur years after primary tumour resection, indicating that disseminated tumour cells can sustain a prolonged quiescent state in distant organs [94]. In tissues such as the bone marrow, the microenvironment secretes factors like bone morphogenetic protein 7 and transforming growth factor‐beta, which activate the p38 mitogen‐activated protein kinase pathway in disseminated tumour cells. This activation upregulates cell cycle inhibitors such as p27, prompting these cells to enter or maintain dormancy [257, 258, 259, 260].

Dormancy maintenance strategies aim to reinforce these endogenous inhibitory signals. For example, disseminated tumour cells with high Axl expression often exhibit enhanced stemness and a propensity for dormancy [261, 262]. Pharmacologically stabilizing the dormancy niche or maintaining a quiescent microvascular environment to prevent the angiogenic switch could theoretically prolong DTC latency and reduce the risk of explosive metastasis [263, 264]. However, this approach requires long‐term safety monitoring and careful assessment of the balance between sustained dormancy and the risk of reactivation [99].

#### 7.3.2 The Awaken and Kill Strategy: Exploiting Cell Cycle Vulnerabilities

Traditional chemotherapy has limited efficacy against dormant, low‐metabolism disseminated tumour cells, making metastasis exceptionally difficult to eradicate [265, 266]. The awaken and kill strategy attempts to first induce disseminated tumour cells to exit dormancy and enter the proliferative phase, followed by clearance using cell‐cycle‐specific therapeutics [104, 267]. Various factors within the pre‐metastatic niche can trigger DTC reactivation, including neutrophil extracellular traps and angiogenic signals. Proteases released by neutrophil extracellular traps, such as neutrophil elastase and matrix metalloproteinase 9, cleave laminin to generate specific fragments that activate α3β1 integrins on disseminated tumour cells, thereby initiating proliferative signalling [115, 268]. Furthermore, when stable microvessels transition to active angiogenesis, endothelial‐derived signals can also induce DTC cell cycle re‐entry [256, 269]. Crucially, the clinical translation of this strategy demands extreme temporal precision: failing to effectively eliminate disseminated tumour cells during their most sensitive vulnerability window could paradoxically accelerate metastatic progression [268].

Despite its strong conceptual appeal, the ‘awaken and kill’ paradigm represents a double‐edged sword fraught with severe clinical hazards. The paramount danger lies in incomplete therapeutic eradication: if the secondary cytotoxic or immunotherapeutic regimen fails to eliminate 100% of the newly awakened DTCs, this strategy risks paradoxically accelerating explosive metastatic outgrowth and precipitating rapid disease recurrence [268, 270, 271]. To safely translate this high‐risk strategy into clinical practice, three strict preconditions and risk‐mitigation frameworks must be established:

*Strict patient stratification and biomarker preconditions*: This strategy is strictly contraindicated in patients with high tumour burden or unmonitored latent disease. It should be restricted exclusively to minimal residual disease (MRD) settings where DTC dormancy state and reactivation kinetics can be dynamically tracked via high‐sensitivity liquid biopsies (e.g., monitoring circulating tumour DNA dynamics, exosomal markers, or single‐cell p38/ERK signalling ratios) [92, 271, 272].

*Spatiotemporal synchrony via smart nanomedicine*: To prevent therapeutic time lags where DTCs escape clearance after entering the active cell cycle, dual‐responsive nanocarriers or co‐delivery systems should be deployed. These smart platforms ensure that dormancy‐awakening agents (e.g., NET inhibitors or ERK pathway activators) and potent cell‐cycle‐specific cytotoxic agents (or targeted immunotherapies) are released in precise, synchronized spatiotemporal windows within the pre‐metastatic niche [273, 274, 275].

*Synergistic immunological backup* (*synthetic lethality*): Rather than relying solely on conventional chemotherapy, awakening agents should be combined with immune checkpoint blockade (e.g., anti‐PD‐1/PD‐L1) or CAR‐T cell therapies. Re‐entering the cell cycle frequently triggers cell surface antigen exposure and immunogenic cell death pathways, allowing host effector T cells to serve as a biological ‘safety net’ to systematically hunt and eliminate any residual proliferating clones [276, 277, 278, 279].

By integrating real‐time MRD monitoring, spatiotemporal drug co‐delivery and synergistic immune sensitization, the inherent hazards of DTC awakening can be effectively neutralized, transforming a hazardous therapeutic concept into a controllable clinical intervention.

#### 7.3.3 Chronobiology and Chronotherapy in Metastasis Prevention

Beyond its practical application in optimizing therapeutic timing, chronobiology plays a fundamental role in governing host physiological homeostasis and target organ susceptibility to remote education. Circadian disruption, driven by environmental stress, sleep fragmentation, shift work, or cell‐autonomous clock gene alterations (e.g., *BMAL1*, *CLOCK*, *PER2*), acts as a potent upstream orchestrator that actively accelerates PMN initiation and priming:

*Neuroendocrine dysregulation and systemic inflammation*: Circadian desynchrony suppresses nocturnal melatonin secretion while inducing aberrant, sustained spikes in systemic glucocorticoids. This blunted hormonal rhythm impairs systemic immune surveillance and fuels chronic, low‐grade systemic inflammation, facilitating remote tissue priming [280, 281].

*Rhythmic endothelial leakiness and EV secretion*: Tumour cell‐autonomous clock gene blunting significantly augments EV biogenesis and pro‐metastatic cargo loading during specific diurnal windows. Concurrently, diurnal variations in endothelial tight junction turnover mean that circadian desynchrony escalates vascular hyperpermeability in target organs, opening spatial conduits for circulating factor deposition [282, 283, 284].

*Temporal immune cell trafficking and NETosis*: The trafficking of bone marrow‐derived immune cells follows a strict physiological circadian rhythm. Upstream circadian disruption causes aberrant, uncoordinated mobilization and accumulation of immunosuppressive PMN‐MDSCs and neutrophils in distant tissues. Furthermore, blunted clock gene expression lowers the threshold for neutrophil extracellular trap formation, creating localized inflammatory scaffolds that prime distant tissue microenvironments prior to DTC arrival [280, 285, 286].

Building upon these upstream mechanisms, the clinical application of chronotherapy in metastasis prevention has recently gained significant attention. Studies reveal that circulating tumour cell release and DTC colonization within the pre‐metastatic niche follow a circadian rhythm, with peak risks often aligning with the host's resting phase [287]. This rhythm is modulated by systemic factors such as melatonin and glucocorticoids, which subsequently influence cytokine secretion and immune cell activity within the pre‐metastatic niche [288, 289, 290, 291]. Chronotherapy advocates optimizing drug administration timing based on these physiological rhythms. For instance, administering EV inhibitors before peak CTC release or delivering immunomodulators when pre‐metastatic niche priming signals are strongest may achieve superior inhibition of metastasis at lower doses [292]. Moreover, the circadian influence on DTC cell cycle transitions provides a precise temporal coordinate for optimizing the awaken and kill strategy [287, 293].

### 7.4 Microenvironment Normalization: Shifting From Cytotoxicity to Homeostatic Reshaping

The microenvironment normalization strategy marks a significant paradigm shift in anti‐metastatic therapy, positing that metastasis stems from ecological imbalances in distant organs rather than from isolated tumour cell expansion [239, 294]. Restoring the pathological pre‐metastatic niche toward physiological homeostasis through physical, chemical, or biological interventions can fundamentally deprive disseminating cells of essential microenvironmental support [295, 296, 297].

First, vascular normalization optimizes oxygenation and drug delivery. Pre‐metastatic niche vessels are characteristically disorganized, hyperpermeable and deficient in pericytes, which elevates interstitial fluid pressure and exacerbates hypoxia [296]. This hypoxic state drives M2‐like macrophage polarization, EMT and DTC reactivation [298, 299].

Instead of complete vascular ablation, normalization uses judicious doses of anti‐angiogenic agents, such as anti‐vascular endothelial growth factor antibodies, to repair vessel architecture. This reduces interstitial hydrostatic pressure and restores the transcapillary gradient, thereby enhancing the delivery of systemic chemotherapeutics and immune cells into the pre‐metastatic niche [239]. Second, alleviating the physical constraints of the pre‐metastatic niche, specifically matrix stiffness and solid stress, is crucial for reversing cell proliferation and immune exclusion [300, 301]. Abundant collagen and hyaluronic acid deposition by cancer‐associated fibroblasts stiffens the extracellular matrix, triggering integrin‐FAK and YAP/TAZ mechanotransduction. Targeted interventions include using losartan to block angiotensin signalling, thereby reducing matrix synthesis and solid stress; administering collagenase or hyaluronidase to dismantle physical barriers; or employing FAK or YAP inhibitors to disrupt stiffness‐mediated mitogenic pathways [70, 302, 303].

Rather than serving as a standalone modality, microenvironment normalization functions primarily as an ecological sensitizer. For example, combining vascular normalization with immune checkpoint inhibitors consistently outperforms monotherapies [239, 304]. This synergy relies on a normalized microenvironment to diminish local accumulation of immunosuppressive factors and overcome physical barriers to T‐cell infiltration [296, 305]. Future paradigms point toward smart drug delivery systems designed for real‐time microenvironmental sensing. Multi‐stimuli‐responsive nanoparticles could achieve spatiotemporally controlled release of anti‐platelet agents, cytotoxic drugs, or immunomodulators, triggered by the acidic pH or upregulated matrix metalloproteinase levels characteristic of the pre‐metastatic niche [275, 306, 307]. Such integrated regimens offer a promising avenue to address metastatic heterogeneity and advance the therapeutic window before the emergence of macro‐metastases.

Once disseminated tumour cells reach the pre‐metastatic niche, their fate is tightly regulated by microenvironmental signals. The re‐entry of these cells from G0‐phase dormancy into the active cell cycle represents the critical rate‐limiting step in the progression from micrometastases to overt metastatic lesions [267]. Consequently, therapeutic strategies diverge into two complementary directions: maintaining dormancy to delay or prevent metastasis, or inducing DTC proliferation followed by cell‐cycle‐specific eradication [267, 268].

#### 7.4.1 Maintaining Dormancy: Strategies to Delay Metastatic Progression

Metastatic relapse can occur years after primary tumour resection, indicating that disseminated tumour cells can sustain a prolonged quiescent state in distant organs. In tissues such as the bone marrow, the microenvironment secretes factors like bone morphogenetic protein 7 and transforming growth factor‐beta, which activate the p38 mitogen‐activated protein kinase pathway in disseminated tumour cells [258]. This activation upregulates cell cycle inhibitors such as p27, prompting these cells to enter or maintain dormancy [258].

Dormancy maintenance strategies aim to reinforce these endogenous inhibitory signals. For example, disseminated tumour cells with high Axl expression often exhibit enhanced stemness and a propensity for dormancy [261]. Pharmacologically stabilizing the dormancy niche or maintaining a quiescent microvascular environment to prevent the angiogenic switch could theoretically prolong DTC latency and reduce the risk of explosive metastasis [256]. However, this approach requires long‐term safety monitoring and careful assessment of the balance between sustained dormancy and the risk of reactivation [115].

#### 7.4.2 The Awaken and Kill Strategy: Exploiting Cell Cycle Vulnerabilities

Traditional chemotherapy has limited efficacy against dormant, low‐metabolism disseminated tumour cells, making metastasis exceptionally difficult to eradicate. The awaken and kill strategy attempts to first induce disseminated tumour cells to exit dormancy and enter the proliferative phase, followed by clearance using cell‐cycle‐specific therapeutics [267]. Various factors within the pre‐metastatic niche can trigger DTC reactivation, including neutrophil extracellular traps and angiogenic signals. Proteases released by neutrophil extracellular traps, such as neutrophil elastase and matrix metalloproteinase 9, cleave laminin to generate specific fragments that activate α3β1 integrins on disseminated tumour cells, thereby initiating proliferative signalling [115]. Furthermore, when stable microvessels transition to active angiogenesis, endothelial‐derived signals can also induce DTC cell cycle re‐entry [256]. Crucially, the clinical translation of this strategy demands extreme temporal precision: failing to effectively eliminate disseminated tumour cells during their most sensitive vulnerability window could paradoxically accelerate metastatic progression [268].

#### 7.4.3 Chronobiology and Chronotherapy in Metastasis Prevention

Recently, the circadian regulation of tumour metastasis has gained significant attention [308]. Studies reveal that circulating tumour cell release and DTC colonization within the pre‐metastatic niche follow a circadian rhythm, with peak risks often aligning with the host's resting phase [287]. This rhythm is modulated by systemic factors such as melatonin and glucocorticoids, which subsequently influence cytokine secretion and immune cell activity within the pre‐metastatic niche [309]. Chronotherapy advocates optimizing drug administration timing based on these physiological rhythms [310, 311, 312]. For instance, administering EV inhibitors before peak CTC release, or delivering immunomodulators when pre‐metastatic niche priming signals are strongest, may achieve superior inhibition of metastasis at lower doses [313]. Moreover, the circadian influence on DTC cell cycle transitions provides a precise temporal coordinate for optimizing the awaken and kill strategy [310].

### 7.5 Microenvironment Normalization: Shifting From Cytotoxicity to Homeostatic Reshaping

The microenvironment normalization strategy marks a significant paradigm shift in anti‐metastatic therapy, positing that metastasis stems from ecological imbalances in distant organs rather than from isolated tumour cell expansion. Restoring the pathological pre‐metastatic niche toward physiological homeostasis through physical, chemical, or biological interventions can fundamentally deprive disseminating cells of essential microenvironmental support [314].

#### 7.5.1 Physical and Vascular Normalization Pathways

PMN vessels are characteristically disorganized, hyperpermeable and deficient in pericytes, which elevates interstitial fluid pressure and exacerbates hypoxia. Instead of complete vascular ablation, normalization uses judicious doses of anti‐angiogenic agents (e.g., low‐dose anti‐VEGF antibodies) to repair vessel architecture, reducing interstitial hydrostatic pressure and restoring transcapillary gradients to enhance systemic drug and immune cell delivery [137, 239, 315, 316, 317]. Concurrently, alleviating physical constraints, specifically matrix stiffness and solid stress, is crucial for reversing cell proliferation and immune exclusion. Targeted interventions include using losartan to block angiotensin signalling (reducing matrix synthesis), administering hyaluronidase/collagenase to dismantle physical barriers or employing FAK and YAP inhibitors to disrupt stiffness‐mediated mechanotransduction [301, 318, 319, 320]. Rather than serving as a standalone modality, microenvironment normalization functions primarily as an ecological sensitizer, synergizing with immune checkpoint inhibitors to diminish local accumulation of immunosuppressive factors and overcome physical barriers to T‐cell infiltration.

#### 7.5.2 Liquid Biopsy‐Derived PMN Biomarkers and Clinical Cohort Validation

The clinical translation of PMN‐targeted interventions fundamentally hinges on our ability to non‐invasively detect pre‐metastatic tissue priming before overt macrometastases emerge. Liquid biopsy platforms, capturing tumour‐derived EVs, circulating tumour DNA, tumour‐educated platelets and soluble inflammatory mediators, have emerged as crucial non‐invasive tools for PMN risk stratification:

*Exosomal cargo and surface markers*: EV‐bound integrins (e.g., exosomal α_v_β_5_ for liver tropism and α_6_β_4_ for lung tropism) and non‐coding RNAs (e.g., miR‐21, miR‐122‐5p) serve as functional zip codes for PMN priming. Prospective clinical cohorts have validated that elevated pre‐operative plasma levels of exosomal PD‐L1 and S100A8/A9 correlate strongly with early visceral recurrence and immunosuppressive PMN formation in breast and lung cancer patients [11, 140, 321].

*Circulating soluble factors and myeloid mobilization signals*: Elevated circulating levels of LOX, S100A8/A9, VEGF‐A and G‐CSF have been prospectively validated in clinical cohorts as early predictive indicators of distant metastatic relapse. For instance, high plasma LOX activity in early‐stage breast cancer patients independently predicts subsequent bone microenvironment remodelling and osteolytic relapse [51, 322, 323].

*TEPs and ctDNA dynamics*: TEPs sequester tumour‐derived RNAs and cytokines during circulation, offering a dynamic fingerprint of systemic PMN priming. In prospective observational studies, integrated panel testing combining ctDNA clearance dynamics with TEP gene signatures significantly improved the predictive accuracy for early metastatic recurrence compared to ctDNA alone [324].

#### 7.5.3 Off‐Target Toxicity Risks and Safety Considerations in Long‐Term Interventions

Because PMN normalization strategies target host stromal, endothelial and immune compartments rather than tumour‐intrinsic mutations, long‐term administration carries non‐negligible risks of off‐target toxicity that must be critically managed:

*Stromal and ECM‐modifying agents*: LOX inhibitors (e.g., BAPN, Simtuzumab) effectively reduce extracellular matrix stiffness; however, systemically inhibiting LOX disrupts physiological collagen crosslinking in healthy vascular walls and cartilage, posing risks of arterial aneurysms and musculoskeletal pain. Preclinical safety studies indicate that pulsed or intermittent dosing schedules can preserve vascular integrity while maintaining therapeutic anti‐fibrotic efficacy in target organs [325, 326].

*Myeloid and macrophage depletion/reprogramming*: Long‐term inhibition of CSF‐1R (e.g., Pexidartinib) or CCR2/CCR5 (e.g., BMS‐813160) effectively dismantles PMN immunosuppressive networks. However, systemic macrophage depletion frequently leads to facial oedema, liver toxicity (transaminitis) and increased susceptibility to opportunistic infections due to the loss of tissue‐resident physiological macrophages (e.g., Kupffer cells, alveolar macrophages) [327, 328, 329].

*Vascular normalization modalities*: While low‐dose anti‐VEGF therapy repairs hyperpermeable PMN microvessels, prolonged anti‐angiogenic pressure can paradoxically aggravate systemic hypertension, delayed wound healing and local tissue hypoxia, potentially driving secondary EMT and therapy resistance [315, 330, 331, 332].

#### 7.5.4 Clinical Translation: Evidence From Trials Evaluating PMN Stratification, Chronotherapy and Nanocarriers

To bridge basic mechanistic discoveries with clinical oncology, several clinical trials are actively evaluating PMN risk stratification, chronotherapy optimization and microenvironment‐responsive delivery systems:

*Liquid biopsy‐guided PMN risk stratification*: The DYNAMIC trial (NCT03088150) established ctDNA‐guided risk stratification as a clinically valid strategy to tailor post‐operative therapy in stage II colon cancer [333]. Extending this paradigm to PMN‐specific analytes, emerging evidence supports EV‐RNA panels and EV‐associated S100A8/A9 as candidate biomarkers of niche priming. EV‐RNA signatures have shown pooled sensitivity and specificity of 82% and 79% for colorectal cancer detection and circulating S100A9‐bearing EVs correlate with the histopathology and immunophenotype of colorectal liver metastases [334, 335]. A prospective study (NCT07754513) is now evaluating EV‐derived biomarkers in metastatic colorectal cancer. However, no published prospective trial has yet demonstrated that integrated EV‐RNA/S100A8/A9 panels can stratify patients for pre‐emptive PMN‐directed therapy before radiological metastasis detection. Current efforts remain in analytical and early clinical validation, requiring standardized PMN activation biomarkers, risk‐based enrolment and endpoints linking niche modulation to recurrence‐free survival.

*PMN‐directed targeted agents in clinical trials*: Multiple agents targeting PMN priming components have advanced to Phase I/II clinical evaluation. These include FAK inhibitors (Defactinib/VS‐6063, NCT02546531) combined with immune checkpoint inhibitors to alleviate stiffness‐induced immune exclusion [336], CCR2/CCR5 dual antagonists (BMS‐813160, NCT03184870) to block MDSC mobilization to distant organs [337], CXCR4 antagonists (Motixafortide/BL‐8040, NCT03277209) to disrupt the PMN retention niche [338] and CSF‐1R inhibitors (Pexidartinib, NCT02452424) to reprogram M2‐like TAMs.

*Chronotherapy dosing optimization in the clinic*: The phase III LungTIME‐C01 trial (NCT05549037) reported improved progression‐free survival (11.3 vs. 5.7 months; HR 0.40) and overall survival (28.0 vs. 16.8 months; HR 0.42) with early‐day chemo‐immunotherapy in advanced NSCLC, but has since been retracted. Ongoing phase IV MELCHRONO (NCT07574047) and phase II TIME trials are testing discrete morning, midday and afternoon infusion windows in melanoma [339]. Preclinical evidence supports time‐adapted anti‐angiogenic dosing based on circadian VEGF expression [340], while chemotherapy chronotherapy trials (CHRONOFOX and EORTC 05962) demonstrate reduced cardiotoxicity and nephrotoxicity with chronomodulation [341, 342]. Mechanistically, Bmal1–CXCL2–CXCR2 signalling drives neutrophil egress [343] and NETosis peaks in the early morning (~8 AM in humans), suggesting that avoiding this window may limit PMN priming [285]. However, no prospective trial has yet shown reduced PMN formation or improved metastasis‐free survival and the LungTIME‐C01 retraction underscores the need for validated circadian biomarkers and adequately powered confirmatory trials.

*Stimulus‐responsive nanocarrier delivery systems*: Advanced nanomedicine platforms engineered for PMN‐specific environmental triggers (such as elevated matrix metalloproteinase levels or acidic pH) are undergoing clinical evaluation. For example, MMP‐cleavable and pH‐responsive nanoparticles (e.g., BIND‐014, NC‐6004) ensure targeted drug accumulation specifically within primed pre‐metastatic tissues, effectively minimizing off‐target organ toxicity while maximizing local drug [344, 345, 346] (summarized in Table 6).

**TABLE 6:** Summary of candidate liquid biopsy‐derived PMN biomarkers and representative clinical trials of PMN‐targeted therapeutics.

Category	Candidate biomarkers/targeted agents	Target PMN mechanism	Clinical validation status/trial identifiers	References
Liquid biopsy biomarkers	Exosomal integrins (α_v_β_5_, α_6_β_4_)	EV organotropism and vascular priming	Validated in prospective cohort studies; predictive of organ‐specific recurrence.	[11]
	Plasma S100A8/A9 and LOX activity	Myeloid recruitment and ECM stiffening	Validated in clinical cohorts: LOX in early breast cancer; S100A8/A9 in inflammatory breast cancer (prospective cohort).	[322, 347]
	ctDNA clearance + TEP RNA panels	Systemic PMN priming	ctDNA clearance: DYNAMIC trial (NCT03088150); TEP + ctDNA panels: prospective evaluation ongoing (e.g., NCT04022863, NCT07754513).	[333, 335]
PMN‐targeted clinical agents	Defactinib (VS‐6063, FAK Inhibitor)	Block ECM stiffness mechanotransduction	Phase I/II clinical trials in combination with ICIs (NCT02546531).	[336]
	BMS‐813160 (CCR2/CCR5 Antagonist)	Block MDSC recruitment to distant sites	Phase I/II clinical evaluation in PDAC and CRC metastasis (NCT03184870).	[337]
	Motixafortide (BL‐8040, CXCR4 Antagonist)	Disrupt stromal sanctuary and immune exclusion	Phase II COMBAT trial (NCT02907099) demonstrated enhanced T‐cell infiltration.	[338]
	Pexidartinib (CSF‐1R Inhibitor)	Deplete/Reprogram M2‐like TAMs	Phase I/II clinical trials in solid tumours (NCT02452424).	[348]

## 8 Conclusion and Future Perspectives

The introduction of the pre‐metastatic niche concept has revolutionized the passive understanding of target organs inherent in the classical seed and soil hypothesis, revealing a dynamic process in which primary tumours remotely remodel distant microenvironments through systemic signalling. From the perspectives of systems biology and clinical translation, this review integrates the key characteristics of the pre‐metastatic niche as a multicellular, collaborative proliferative network and proposes a potential pathway to shift anti‐metastatic strategies from reactive treatment to proactive prevention.

### 8.1 Integrating the Pre‐Metastatic Niche Proliferative Ecosystem

Rather than being a random accumulation of cells, the pre‐metastatic niche represents a highly organized, self‐reinforcing multicellular ecosystem. Its evolution can be conceptualized as three distinct phases: the permissive phase, during which tumour‐derived extracellular vesicles and soluble factors such as vascular endothelial growth factor alter the baseline state of the target organ and increase vascular permeability; the instructive phase, in which exosomal molecular zip codes drive the transformation of fibroblasts into cancer‐associated fibroblasts, recruit myeloid‐derived suppressor cells and induce M2‐like macrophage polarization to construct an immune‐privileged microenvironment; and the selective phase, where disseminated tumour cells, acting as a functional readout of the pre‐metastatic niche state, re‐enter the cell cycle from dormancy under conditions of increased metabolic availability and altered matrix stiffness, ultimately driving metastatic colonization. Spatial multi‐omics research has revealed highly compartmentalized ligand–receptor interaction networks within the pre‐metastatic niche, exemplified by dense communication among malignant, myeloid and endothelial cells surrounding hypoxic necrotic zones. Concurrently, the pre‐metastatic niche exhibits hallmarks of an oncogenic ecosystem: tumour EVs remodel glucose metabolism in target organs; cancer‐associated fibroblasts and myeloid‐derived suppressor cells promote T‐cell exhaustion through altered lipid and amino acid metabolism; and epigenetic modifications fix these pathological states into cellular memories. Furthermore, the extracellular matrix is no longer a passive scaffold but actively regulates EMT, drug resistance and cell proliferation through integrin‐FAK pathways and YAP/TAZ mechanotransduction. Collectively, this evidence demonstrates that the pre‐metastatic niche is a dynamic network governed by multicellular interactions, metabolic resetting, epigenetic regulation and biomechanical signalling.

### 8.2 From Reactive Treatment to Proactive Interception: Translational Realities and Future Horizons

Translating the PMN framework into clinical oncology necessitates a rigorous, evidence‐based distinction between therapies directly validated in distant PMN models and those extrapolated from primary TME studies. Currently, PMN‐directed concepts exist across a wide spectrum of translational readiness. Targeted agents that block distant immune recruitment, such as CXCR4 antagonists (e.g., Motixafortide), dual CCR2/CCR5 inhibitors (e.g., BMS‐813160) and CSF‐1R inhibitors (e.g., Pexidartinib), have demonstrated direct efficacy in distant PMN models and advanced into active Phase I/II clinical evaluation. In contrast, prominent microenvironment normalization strategies, including low‐dose anti‐angiogenic therapy for vascular normalization and losartan‐mediated angiotensin blockade for matrix decompression, have established clinical safety and anti‐stromal efficacy primarily within primary tumour microenvironments. While these approaches offer compelling theoretical rationales for normalizing distant pre‐metastatic soil, direct evidence establishing their efficacy, therapeutic dosing windows and clinical benefit specifically in distant pre‐metastatic niches remains uncertain and warrants dedicated preclinical PMN validation. Beneath these clinically evaluated approaches lies a robust tier of preclinically validated targets; interventions including EV secretion inhibitors (e.g., GW4869 analogues), LOX crosslinking blockers and NETosis inhibitors (e.g., DNase I and PAD4 inhibitors) demonstrate compelling anti‐metastatic efficacy in animal PMN models, yet currently lack human clinical validation. Finally, highly innovative paradigms such as multi‐analyte liquid‐biopsy ‘dynamic metastatic mapping’ (combining EV‐bound integrins, CTC phenotypes and tumour‐educated platelets), AI‐driven digital twin modelling for predicting niche evolution and stimulus‐responsive smart nanocarriers represent exploratory, hypothesis‐generating concepts that remain strictly investigational at present.

Bridging the gap between these conceptual models and clinical implementation entails navigating several formidable clinical, diagnostic and ethical hurdles. Chief among these is patient selection and the risk of overtreatment; because PMN priming occurs in radiologically occult settings, administering systemic microenvironment‐modifying therapies without precise risk stratification risks exposing patients who may never progress to macrometastasis to unnecessary toxicities. This issue is inextricably linked to determining the optimal timing and window of intervention, as initiating therapy too late fails to prevent DTC seeding, whereas premature intervention without clear biological indicators complicates overall clinical management. From a diagnostic standpoint, candidate non‐invasive biomarkers, such as EV‐RNA panels or tumour‐educated platelet signatures, require extensive multi‐centre prospective validation and CLIA‐certified standardization before achieving companion diagnostic utility. Furthermore, the requirement for long‐term administration raises critical concerns regarding treatment duration and cumulative off‐target toxicity, as sustained suppression of host stromal or immune compartments (e.g., chronic LOX inhibition, systemic macrophage depletion, or prolonged anti‐vascular pressure) can disrupt physiological tissue homeostasis and trigger severe adverse effects, such as arterial aneurysms, transaminitis, or secondary hypoxia‐driven therapy resistance. Finally, evaluating PMN interventions presents a unique regulatory bottleneck regarding clinical trial endpoints: because standard RECIST criteria cannot assess non‐visible pre‐metastatic tissue changes, clinical trial paradigms must establish and validate surrogate endpoints, such as ctDNA/EV clearance kinetics or disease‐free survival prolongation within MRD cohorts, to definitively demonstrate therapeutic benefit. Addressing these multifaceted barriers through liquid‐biopsy‐guided, time‐limited trial designs will be essential to safely advance PMN interception from theoretical hypothesis to clinical practice. (summarized in Table 7).

**TABLE 7:** Evidence‐based categorization of PMN diagnostic and therapeutic modalities and their primary translational barriers.

Category/paradigm	Representative modalities/concepts	Model validation status and evidence base	Primary translational barriers	References
PMN‐Validated Clinical Agents	BMS‐813160 (CCR2/5 antagonist), Motixafortide (CXCR4 antagonist), Pexidartinib (CSF‐1R inhibitor)	Validated in preclinical distant PMN models; currently in Phase I/II clinical trials.	Patient selection, defining optimal treatment duration, managing systemic immunosuppression/liver toxicity.	[242, 246, 254]
Extrapolated TME normalization strategies	Low‐dose anti‐VEGF (Vascular normalization), Losartan (Matrix decompression/YAP‐TAZ inhibition)	Extensively validated in primary TME; direct evidence, dosing windows and benefit in distant PMNs remain hypothetical.	Lack of direct PMN dosing window data, risk of systemic hypotension, potential secondary hypoxia/EMT in distant tissues.	[239, 301, 304, 349]
Preclinically validated PMN approaches	GW4869 (EV biogenesis inhibitor), LOX crosslinking inhibitors (BAPN), DNase I/PAD4 inhibitors (NETosis blockers)	Validated in preclinical distant PMN animal models; lacking human clinical trial data.	Lack of human safety profiles, non‐specific organ accumulation, potential impairment of physiological tissue remodelling.	[207, 209, 244, 248]
Hypothesis‐generating and future concepts	Dynamic metastatic mapping (EVs, CTCs, TEPs), AI/digital twin predictive models, stimulus‐responsive smart nanocarriers	Conceptual frameworks, early‐stage exploratory computational/pre‐clinical modelling	Lack of prospective CLIA biomarker validation, algorithm overfitting, complex regulatory approval pathways for AI/nanomedicine	[82, 275, 291, 350]

## Author Contributions

Shengqian Wang, Chenghu Song and Pengfei Li contributed equally to the primary conception and design of the work. Junjie Feng, Xiaoxuan Shi, Qiwen Lu, Bohan Duan, Xinyan Zhang, Siqi Li, Yang Luo, Xiaohui Fan, Ziyun Li, Yang Zhang, Renjun Gu and Wenjun Mao contributed substantially to the acquisition, analysis and interpretation of data. All authors (Shengqian Wang, Chenghu Song, Pengfei Li, Junjie Feng, Xiaoxuan Shi, Qiwen Lu, Bohan Duan, Xinyan Zhang, Siqi Li, Yang Luo, Xiaohui Fan, Ziyun Li, Yang Zhang, Renjun Gu and Wenjun Mao) participated in drafting the manuscript or revising it critically for important intellectual content. All authors provided final approval of the version to be published in Maniscript.docx. All authors agree to be accountable for all aspects of the work, ensuring that questions related to the accuracy or integrity of any part of the research are appropriately investigated and resolved.

## Funding

This work was supported by Natural Science Foundation of Jiangsu Province (BK20240738); Natural Science Research General Program of Jiangsu Higher Education Institutions (24KJB360004); Youth Talent Project of Jiangsu Province Traditional Chinese Medicine Science and Technology Development Plan (QN202206); Luo Linxiu Faculty Development Fund of Nanjing University of Chinese Medicine (LLX202310); and Natural Science Foundation Project of Nanjing University of Chinese Medicine (XZR2024010).

## Conflicts of Interest

The authors declare no conflicts of interest.

## Data Availability

The data that support the findings of this study are available from the corresponding author upon reasonable request.
